# A palette of fluorophores that are differentially accumulated by wild-type and mutant strains of *Escherichia coli*: surrogate ligands for profiling bacterial membrane transporters

**DOI:** 10.1099/mic.0.001016

**Published:** 2021-01-06

**Authors:** Jesus Enrique Salcedo-Sora, Srijan Jindal, Steve O'Hagan, Douglas B. Kell

**Affiliations:** ^1^​ Department of Biochemistry and Systems Biology, Institute of Systems, Molecular and Integrative Biology, Faculty of Health and Life Sciences, University of Liverpool, Crown St, Liverpool L69 7ZB, UK; ^2^​ Department of Chemistry and Manchester Institute of Biotechnology, The University of Manchester, 131 Princess St, Manchester M1 7DN, UK; ^3^​ Novo Nordisk Foundation Centre for Biosustainability, Technical University of Denmark, Building 220, Kemitorvet, 2800 Kgs Lyngby, Denmark

**Keywords:** transporters, bioenergetics, *E. coli*, fluorescence, assays, energization

## Abstract

Our previous work demonstrated that two commonly used fluorescent dyes that were accumulated by wild-type *Escherichia coli* MG1655 were differentially transported in single-gene knockout strains, and also that they might be used as surrogates in flow cytometric transporter assays. We summarize the desirable properties of such stains, and here survey 143 candidate dyes. We eventually triage them (on the basis of signal, accumulation levels and cost) to a palette of 39 commercially available and affordable fluorophores that are accumulated significantly by wild-type cells of the ‘Keio’ strain BW25113, as measured flow cytometrically. Cheminformatic analyses indicate both their similarities and their (much more considerable) structural differences. We describe the effects of pH and of the efflux pump inhibitor chlorpromazine on the accumulation of the dyes. Even the ‘wild-type’ MG1655 and BW25113 strains can differ significantly in their ability to take up such dyes. We illustrate the highly differential uptake of our dyes into strains with particular lesions in, or overexpressed levels of, three particular transporters or transporter components (*yhjV*, *yihN* and *tolC*). The relatively small collection of dyes described offers a rapid, inexpensive, convenient and informative approach to the assessment of microbial physiology and phenotyping of membrane transporter function.

## Introduction

Notwithstanding that the entire genome of strain MG1655 of *Escherichia coli* K12 was sequenced more than 20 years ago [1], some 35 % of its genes are still of unknown function [2]. These genes are known as y-genes [3], and transporters are pre-eminent among them [2]. An important general problem [4, 5] thus involves the related questions of (i) what are the potential substrates for a given transporter, and (ii) what are the membrane transporters for a molecule that cells take up and/or efflux? Our focus here is on finding methods to help provide answers to these questions, in particular for the (approximately) 124 y-genes of *E. coli* that are considered from sequence analysis to be transporters.

Fluorescence flow cytometry provides a convenient and high-throughput means for assessing the extent of uptake and accumulation of a given fluorophore (e.g. [6–18]), and in recent work [19, 20] we have found this to be true in *E. coli in vivo*. In particular, the availability of the Keio collection of single-gene knockouts in ‘non-essential’ genes [21, 22] allowed us to assess the contribution of many of these genes to the uptake of given fluorophores. The chief findings were that the carbocyanine dyes diSC3(5) and SYBR Green I could be taken up and/or effluxed differentially relative to the reference strain by a very great number of strains harbouring single-gene knockouts, consistent with the views that (i) any electron transport-mediated membrane potential was not largely responsible for their steady-state uptake [23–25], and (ii) any non-transporter-mediated transport through the phospholipid bilayer was negligible [26–31].

The fluorophores chosen in this earlier work [20] were two dyes that have been used in the assessment of the numbers and physiological status of intact bacteria, as they seem to permeate wild-type membranes more or less easily (presumably via a variety of transporters). While the strong dependence on transporter activity meant that such dyes did not faithfully reflect either bioenergetic parameters or nucleic acid content, we noted [20] that they opened up the possibility of high-throughput screening of transporter activity, including of competitive or inhibitory (non-fluorescent) substrates of membrane transporters (as in [32] for uptake and [33–38] for efflux). We recognize, of course, that such dyes are unlikely to be the ‘natural’ substrates of these transporters (see also [39]), but that an assessment of differential uptake can provide useful hints as to the kinds of molecular structures with which a given transporter interacts. We recognize too that individual dyes may use multiple transporters, that the number of transporters in *E. coli* significantly exceeds the number of dyes, that multiple dyes may be substrates for the same transporter, and that as with the uptake and efflux of natural substrates we are initially simply seeking an understanding of the activities of transporters of largely unknown potential. Here high-throughput methods come to the fore.

The desirable properties of dyes (and assays) of this type therefore include the following:

1. They are taken up intracellularly more or less rapidly by the target species.
2. They do not interfere significantly with the host’s biochemistry at the concentrations used and on the timescale of interest.
3. They have a high fluorescence signal (usually involving a high absorbance at the excitation wavelength and a high quantum yield).
4. They are not interfered with by autofluorescence (often implying a large Stokes shift or an absorbance nearer the red).
5. They show a fluorescence that is linear with intracellular concentration at the time point(s) of interest (consistent with point 2).
6. Assays should be performed at constant cell number, or if they are not the number of cells used does not materially affect the external concentration of the dye.
7. When used as a surrogate for a particular transporter, the bulk of the flux of interest is mediated by that transporter.

We also recognize that in some cases (not least when binding is to DNA or to metal ions) the binding of such fluorophores to intracellular targets can induce changes in both the magnitude and the fluorescence spectrum of a given dye, or that dyes might exhibit concentration-dependent self-quenching; this will in some cases need to be considered when the uptake of these dyes is used as a surrogate measure for transporter activity. Normally, however, it will be the case that the magnitude of a dye’s specific fluorescence is the same both inside and outside the cell, such that its uptake cannot be registered reliably simply by measuring total fluorescence in a cuvette or a plate reader, but requires the use of an instrument where the signals can be triggered by other means (see also [40]). It goes without saying that the expression of particular transporters (or anything else) depends on the growth conditions used, and these are typically controlled in *E. coli* by transcriptional regulatory networks (e.g. [41–44]). Consequently, in any given condition there is a danger of ‘false negatives’ [45, 46], i.e. rejection of dyes that under other circumstances might be taken up well. Thus, we have sought to be inclusive of potentially useful dyes so far as is possible. We note that neither flow cytometry nor filtration assays can easily discriminate binding from uptake; doing this relies on the use of mutants and other evidence (e.g. where the extent of uptake far exceeds plausible binding sites). We note too that no dye (nor any other substance) is necessarily likely to be a substrate of just a single transporter; again this is established via the use of mutant strains and/or of other conditions varying expression profiles substantially.

**Fig. 1.**
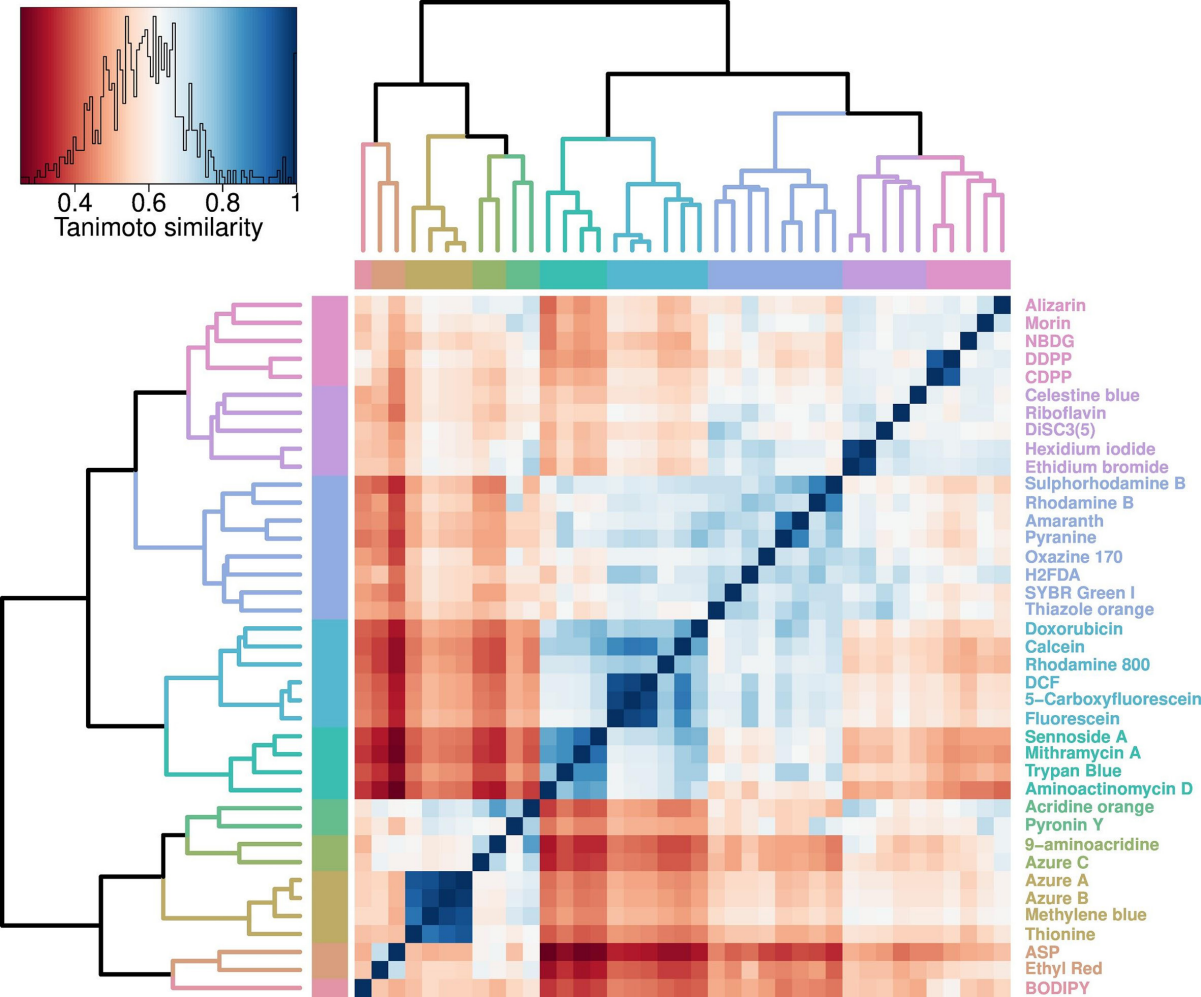
Cheminformatic analysis of fluorophores accumulated by *E. coli* BW25113. Heatmap representing the Tanimoto similarities (derived as described in the Methods section) of the palette of 39 compounds. Scale from zero (least similarity) to 1 (greatest similarity). Stars indicate clusters with a Tanimoto similarity exceeding 0.8 (seen as a cutoff for similar bioactivities).

**Fig. 2.**
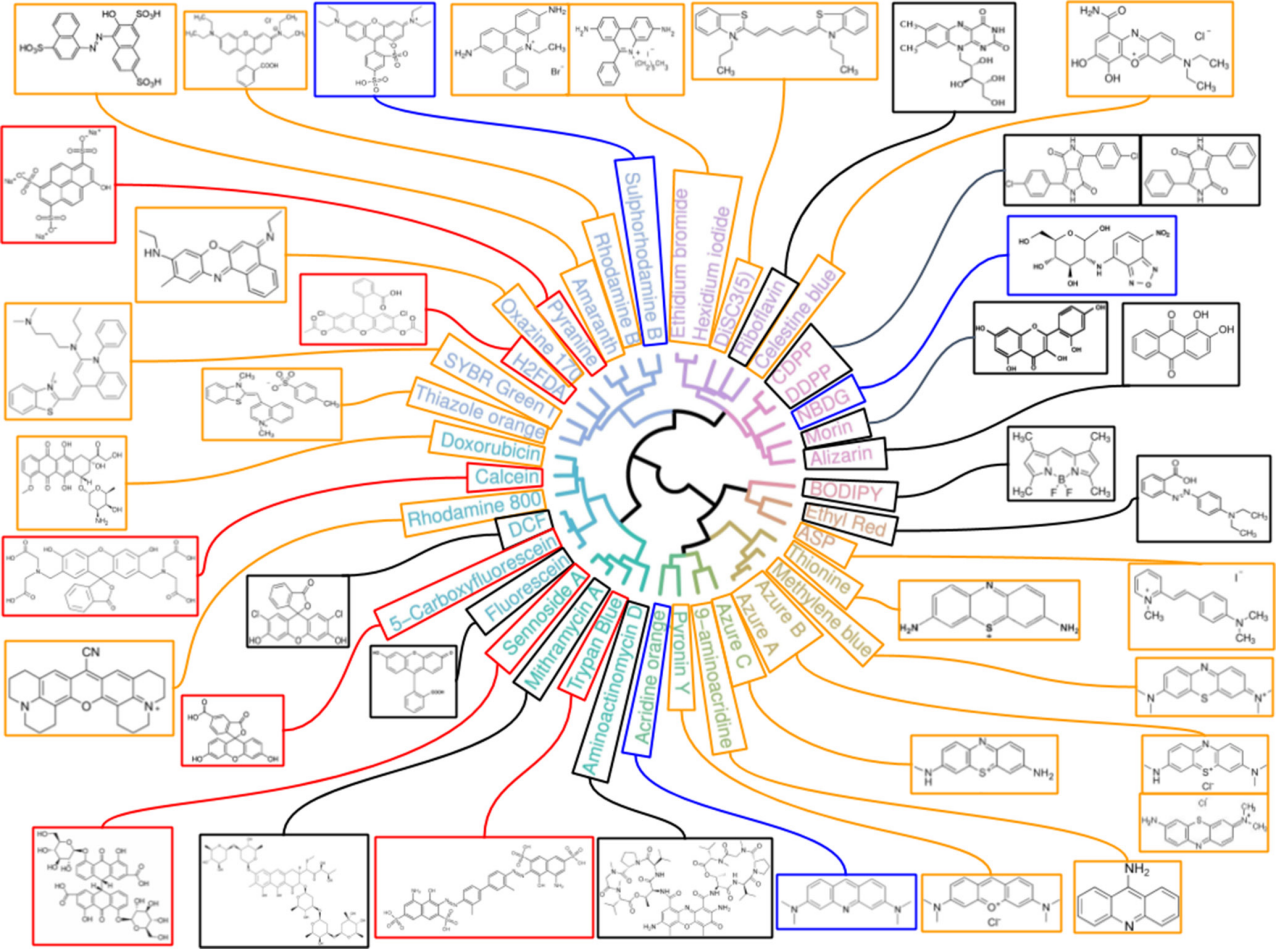
Chemical diversity among 39 fluorophores taken up by *E. coli* BW25113. Circularized topology of the dendrogram in Fig. 1 for the visualization of the structures of this set of fluorophores. The colour of the box indicates the molecular type and/or the expected charge at pH 7.4: black, uncharged; blue, zwitterionic; red, negative; orange, positive.

Dyes with most or all of the desirable properties given above are not particularly common as applied to unfixed cells, and we recognized that a wider survey of potentially useful bacterial membrane-permeating dyes of different structures might prove of considerable value for many purposes. Further, most dyes applied to biological cells are used on mammalian cells, and so may not be pertinent to bacteria. Nonetheless, we surveyed the catalogues of a wide variety of biological stain suppliers, plus other sources such as food colours, laser dyes, dyes used in the water industry for tracking, and even the core scaffolds used in organic light-emitting diodes. Thus, the present study represents an initial survey to this end, and provides a significant number of dyes that do seem to have most or all of the desired properties in the reference strain. We triaged our initial set down to 39 dyes based on a combination of uptake, chemical structure and cost (Fig. 1, Table 1), and these provide our main palette, although if resources are at a premium we also provide a cheminformatics analysis that permits further triaging to as few as six dyes (Fig. 2). In addition, to illustrate their utility in actual cases, we assess their utility in profiling three membrane transporters using appropriate mutants.

**Table 1.** Fluorophore set specifically accumulated by *E. coli* BW25113. List of fluorescent (Fluor) molecules that recorded fluorescence signals two-fold or higher (Signal strength) than the median values of autofluorescence. SMILES (Simplified Molecular Input Line Entry System [135]) Channel (as listed in Methods): Intellicyt® flow cytometer channel that registered the highest median for each fluorophore. The excitation and emission profiles of these channels should have matched the excitation and emission wavelengths for those fluorophores (Table S1). The structures of some of these fluorophores are unknown or unavailable (NA). Concentration: concentration in micromolar at which the highest median values were found for each fluorophore in the range tested. SD: standard deviation of the sample, CI95: 95% confidence interval for the data of signals from each fluorophore at the given channel. BODIPY tetra-methyl: Difluoro{2-[(3,5-dimethyl-2H-pyrrol-2-ylidene-N)methyl]-3,5-dimethyl-1H-pyrrolato-N}boron

Fluor	Channel	Mean	Concentration	Median	SD	CI95	Signal strength
2-(N-(7-Nitrobenz-2-oxa-1,3-diazol-4-yl)Amino)−2-Deoxyglucose	BL3	10 609	2.5	11 615	3311	[9256–11 962]	4.16
2',7′-Dichlorofluorescein	BL3	16 332	3	16 550	4821	[14361–18 302]	5.93
3,6-Bis(4-chlorophenyl)−2,5-dihydropyrrolo[3,4 c]pyrrole-1,4-dione	RL1	12 552	3	12 303	1956	[11695–13 410]	2.92
3,6-Diphenyl-2,5-dihydropyrrolo[3,4 c]pyrrole-1,4-dione	RL1	10 731	3.0	9415	3065	[9388–12 074]	2.24
4-(4-(Dimethylamino)styryl-N-methylpyridinium (ASP+)-	RL1	26 815	0.3	9288	42 128	[17216–36 413]	2.21
5-Carboxyfluorescein	BL3	14 787	1.0	18 608	11 353	[11787–17 788]	6.67
7-Aminoactinomycin D	VL4	11 609	2.5	12 250	2399	[10629–12 589]	2.47
9-aminoacridine	VL1	1458	5.0	1597	426	[1349–1566]	2.83
Acridine orange	BL3	7508	10	7467	3265	[6675–8341]	2.68
Alizarin	BL3	7380	3.0	6862	3767	[6419–8341]	2.46
Amaranth	BL3	6330	5.0	6303	2604	[5665–6994]	2.26
ATTO 430LS carboxy	BL3	5203	5.0	6191	1436	[4616–5790]	2.22
ATTO 488 carboxy	BL3	19 619	1.3	19 556	5804	[18138–21 100]	7.01
ATTO 490LS carboxy	BL5	10 272	3.0	8232	7447	[8046–12 498]	9.66
ATTO 633 carboxy	RL2	20 712	3.0	18 035	11 004	[17423–24 001]	11.1
ATTO 647 carboxy	RL1	46 880	10	38 512	67 804	[29579–64 181]	9.15
ATTO Rho14 carboxy	RL1	30 855	10	12 507	37 080	[19772–41 938]	2.97
Azure A	RL1	10 631	3.0	9994	3830	[9654–11 608]	2.37
Azure B	RL1	11 507	10	10 951	3462	[10623–12 390]	2.60
Azure C	RL1	16 418	2.5	13 675	14 301	[12639–20 197]	3.25
Calcein	BL3	16 131	10	16 616	4493	[14984–17 277]	5.96
Celestine blue	RL1	14 754	10	12 252	7653	[12352–17 156]	2.91
CruzFluor 405 succinimidyl ester	BL3	15 887	10	16 691	4278	[14139–17 636]	5.98
BODIPY tetra-methyl	VL5	7969	1.0	9639	5286	[6310–9628]	2.31
DiSC3(5)	RL1	205 602	1.3	189 686	152 318	[166736–244 468]	45.1
Doxorubicin	BL3	8589	1.0	7583	4062	[7552–9625]	2.72
Ethidium bromide	BL3	11 501	10	11 246	3136	[10701–12 301]	4.03
Ethyl Red	RL1	29 928	10	18 738	25 518	[21920–37 937]	4.45
Fluorescein	BL3	16 869	5.0	17 035	3560	[15961–17 777]	6.11
H2FDA	BL1	9137	5.0	8708	4036	[8107–10 167]	3.54
Hexidium iodide	BL3	12 993	10	13 090	3819	[9250–16 735]	4.69
Methylene blue	RL1	10 259	1.0	9259	4009	[9236–11 282]	2.20
Mithramycin A	VL4	11 137	5.0	11 616	3723	[10187–12 087]	2.34
Morin	BL3	7223	3.0	7437	732	[6506–7940]	2.67
Oxazine 170	RL1	9014	3.0	10 024	3831	[8002–10 026]	2.38
Pyranine	VL4	11 124	10	11 047	6960	[9285–12 964]	2.23
Pyronin Y	BL3	11 652	1.3	9030	6434	[10011–13 294]	3.24
Rhodamine 800	RL2	13 444	1.3	8436	13 241	[9288–17 599]	5.21
Rhodamine B	BL3	7012	1.3	6580	3552	[6106–7918]	2.36
Riboflavin	BL3	7020	1.0	7254	2762	[6315–7724]	2.60
Sennoside A	VL4	9506	10	10 634	2645	[8715–10 297]	2.15
Sulphorhodamine B	BL3	9440	10	8117	4183	[8373–10 508]	2.91
SYBR Green I	BL1	16 690	2.5	8517	19 703	[11663–21 718]	3.46
SYTO13	BL1	281 386	5.0	197 317	235 885	[178007–384 765]	80.1
Thiazole orange	BL1	25 740	10	10 600	32 357	[17484–33 997]	4.30
Thionine	RL1	9878	10	10 166	5068	[8539–11 218]	2.42
Trypan Blue	RL1	19 833	5.0	15 151	16 464	[15482–24 184]	3.60

## Methods

*E. coli* strain BW25113 [Keio collection reference strain: *Δ(araD-araB)567*, *ΔlacZ4787*(::rrnB-3), *λ* ^−^, *rph-1*, *Δ(rhaD-rhaB)568*, *hsdR514*] and certain other representatives of the Keio collection [21, 22] used here included the following gene knockouts: ***yhjV*** [*F-, Δ(araD-araB)567, ΔlacZ4787(::rrnB-3), λ^−^, ΔyhjV722::kan, rph-1, Δ(rhaD-rhaB)568, hsdR514*); ***yihN*** (*F-, Δ(araD-araB)567, ΔlacZ4787(::rrnB-3), λ^−^, rph-1, ΔyihN736::kan, Δ(rhaD-rhaB)568, hsdR514*]; and ***tolC*** [*F-, Δ(araD-araB)567, ΔlacZ4787(::rrnB-3), λ^−^, ΔtolC732::kan, rph-1, Δ(rhaD-rhaB)568, hsdR514*]. We also used the strain overexpressing *yhjV* from the ASKA collection: *E. coli* K-12, strain AG1 [*recA1 endA1 gyrA96 thi-1 hsdR17 (r K− m K+) supE44 relA1*] carrying recombinant constructs in the IPTG-inducible plasmid pCA24N (CmR, *lacIq*). The induction with IPTG was optimized to 250 µM for 3 h (37 °C, shaking at 200 r.p.m.) previous to the fluorophore uptake assays with the ASKA strain. Both the Keio and the ASKA collection (pmid16769691) were provided by the National Institute of Genetics, Mishima, Shizuoka, Japan.

For each fluorophore uptake assay the starting point was the spread of a small flake from a frozen culture stock of *E. coli* onto a fresh plate of complex solid media (Merck LB 110283) containing selective antibiotics when appropriate. Kanamycin was used at 50 µg ml^−1^ final concentration for the Keio knockout strains and chloramphenicol at 30 µg ml^−1^ for the ASKA strain. A single colony from a fresh solid media plate was then incubated in liquid complex media (Merck LB 110285) overnight at 37 °C with shaking at 200 r.p.m. in the absence of antibiotics. The overnight cultures were diluted 1 : 5000 in fresh liquid complex media and grown for 2 h at 37 °C with shaking at 200 r.p.m. The cell density was then adjusted to ~2000 cells µl^−1^, as judged by turbidity (OD_600_). Cells were then exposed to fluorophores (37 °C, 15 min, or as indicated, with shaking at 1300 r.p.m.) in 384-well plates and final volumes of 50 µl.

The experiments with chlorpromazine (CPZ) were performed using two different protocols. For the first set of experiments, the protocol was replicated from a previously described one [20], where 5 µl CPZ (1 mM in DMSO) was added to the wells of a 96-well plate in triplicate. A vacuum centrifuge was used to dry all the DMSO in the plates. Thereafter, 200 µl of overnight-cultured *E. coli* (MG1655 or BW25113) was added to each well at 1000 cells µl^−1^ final concentration in complex media. The plates were sealed and incubated at 30 °C with 900 r.p.m. shaking for 30 min. Then, DiSC3(5) or SYBR Green I were added to a final concentration of 3 µM and 1× (a 10 000-fold dilution of the stock material supplied), respectively [20]. The plate with DiSC3(5) was incubated for 2 min, while the one with SYBR Green I was incubated for 15 min at 37 °C before sampling in the flow cytometer. For the second set of experiments, CPZ was added to overnight-cultured *E. coli* (BW25113; 2000 cells µl^−1^) cells at a final concentration of 10 µM in complex media. This culture was then incubated with different fluorophores (3 µM) at different pH values for 15 min. To modify the pH of the media the following buffers were added individually (cf. [47]) to the media at final concentrations of 100 mM: MES (pH 6, 6.5), MOPS (pH 7.0, 7.5, 8.0) and Bicine (pH 8.5). Fluorophores were purchased from Sigma Aldrich, Thermo Fisher, TCI America, or ATTO-TEC GmbH. Other reagents were purchased from Sigma Aldrich unless stated otherwise.

We used a high-throughput flow cytometer, the Intellicyt iQue Screener Plus (Sartorius, Göttingen, Germany) [19, 20], with the following protocol: buffer equilibration (QSol, Sartorius) and plate shaking at 2000 r.p.m. for 50 s, sampling for 2 s with 1 s upload time, 5 s wash in Qsol buffer every three wells, and further probe wash for 10 s every 12 wells. The instrument has three LED lasers (405 nm, 488 nm, 640 nm) and collects data for 2 light scattering and 13 fluorescence channels. Once the light from any of three lasers has reached the samples, these channels collect the fluorescent signals back from the samples in the following spectral ranges (in nm): VL1 (445±45), VL2 (530±30), VL3 (572±28), VL4 (615±24), VL5 (675±30), VL6 (780±60), BL1 (530±30), BL2 (572±28), BL3 (615±24), BL4 (675±30), BL5 (780±60), RL1(675±30) and RL2(780±60). In contrast to some instruments [40] (and as is evident from the data), this instrument is highly resistant to the detection of extracellular fluorescence. As with any other transport assay method, such as those based on filtration or on flow dialysis [48], we do not directly discriminate molecules that are bound from those that are intracellular; other arguments given below serve to do that.

Data were analysed and displayed using a combination of the instrument’s Forecyt software, FlowJo, and routines written by the first author in R (all scripts available upon request to the first author).For the dose–response regression analyses the data were fitted with a non-parametric approach (locally weighted scatterplot smoothing) as implemented in R’s ggplot2. The hierarchical dendrogram and heatmap of the palette of 39 fluorophores were scripted with R’s *dendextend* and *ggplot*’s *heatmap.2* packages. The *hclust* function of *dendextend* produced hierarchical clusters from a Tanimoto similarity matrix derived as follows: fingerprints of 39 fluorophores were derived from their SMILES [49] using the Patterned algorithm within the RDkit (www.rdkit.org/) nodes in KNIME (http://knime.org/) [39, 50]. Fingerprints are binary strings (a vector of 1 and 0 s), from which a Tanimoto distance can be calculated in terms of the fraction of positions in which the pairwise elements match (e.g. [51–54]). A Tanimoto distance (TD) matrix was generated from these fingerprints (KNIME’s Distance Matrix Calculate); and the scores of this matrix were converted to Tanimoto similarity indices (1-TD) using KNIME’s Similarity Search. In one case, the hierarchical dendrogram was circularized with R’s *circlize* package to allow space for the composite with the structures of these 39 fluorophores.

## Results

### Baseline analysis

**Fig. 3.**
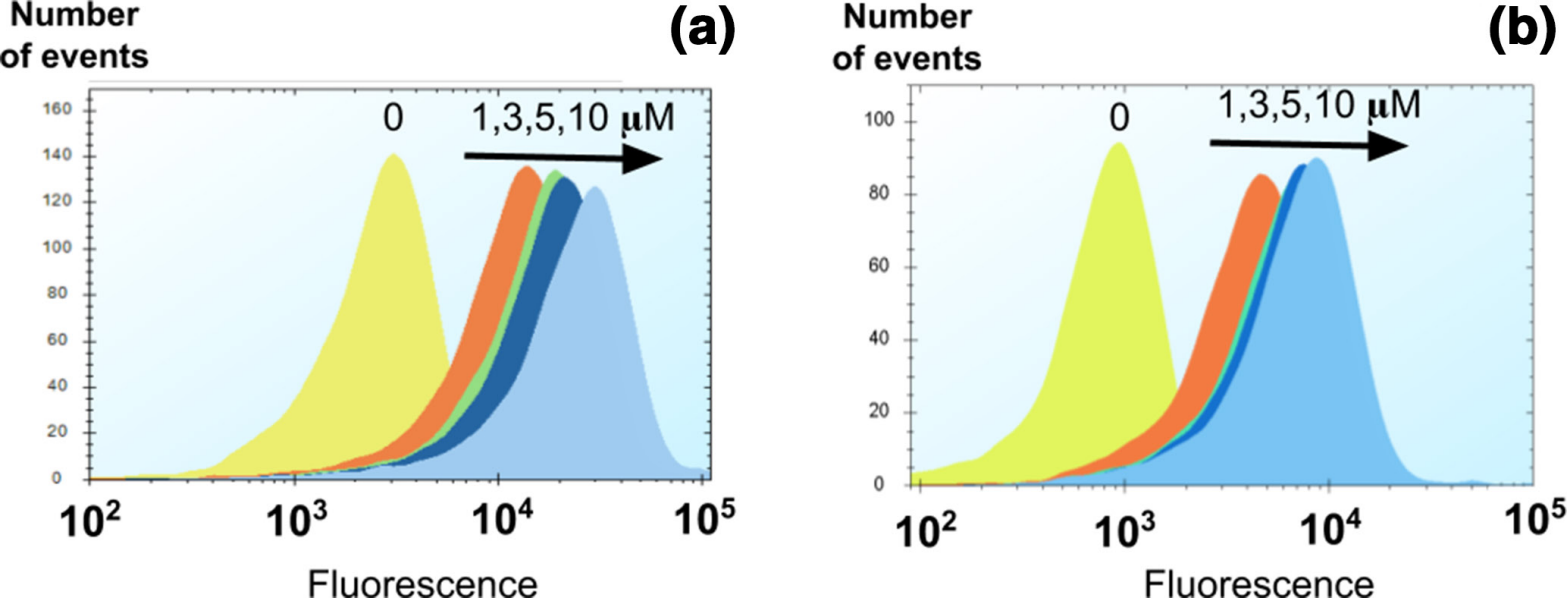
Cytograms of calcein and fluorescein. Cells were grown, harvested and reinoculated at a certain concentration, as described in the Methods section. Both dyes detected in channel BL3 of Intellicyt (ex=488, em=615/24). (a) Calcein fluorescence signals at 1, 3, 5 and 10 µM against the non-dye control (0). (b) Fluorescein signals at 1, 3, 5 and 10 µM against the non-dye control (0).

A total of 143 fluorophores were tested in *E. coli* BW25113 (Table S1, Fig. S1, available in the online version of this article). Fig. 3 shows typical cytograms of stained and unstained cells at four concentrations of two particular dyes, in this case calcein (A) and fluorescein (B). The typical existence of a small population with a very high fluorescence biases the mean and so we normally use the median fluorescence for data analysis.

The fluorescence signals across the entire set of fluorophores spans some three orders of magnitude, with a median value approximately 1×10^3.6^ (Fig. S2a). Autofluorescence (the fluorescence from cells to which no dye has been added) provides a ‘baseline’ (orange line for each channel in Fig. S2b), above which any fluorescence resulting from dye uptake must be resolved (though note that some dyes can act to quench autofluorescence). The channel that captured fluorescence emissions in the region of 530±30 nm (VL2) exhibits the highest values of autofluorescence relative to the other channels (Fig. S2b). The same data after correction for autofluorescence showed a median of approximately 1×10^3.3^ (Fig. S2c). (Note that because dyes can potentially quench autofluorescence, the fluorescence changes induced by dyes include such phenomena.). Also, a fractional carry over between wells was observed in one of the red channels. The latter is observed as a separate cluster at the very highest values in channel RL1 (Fig. S2d). To avoid this, future experiments were designed, and the data filtered, accordingly.

**Fig. 4.**
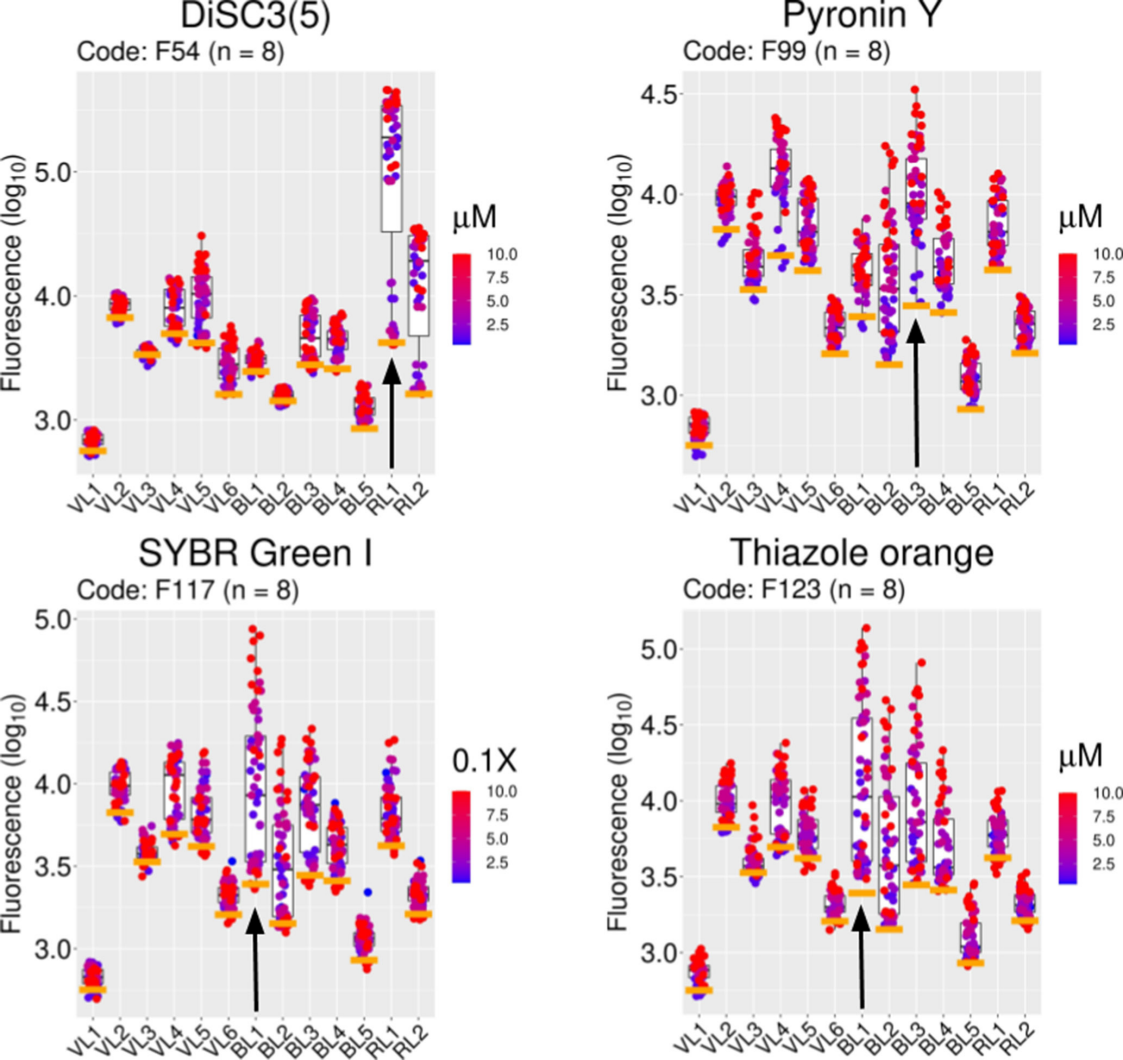
Examples of specific fluorophore uptake in *E. coli* BW25113. Fluorescence signals from 4 fluorophores selected from the set of 47 molecules (Table 1) with values twofold or higher above autofluorescence. The signals for all fluorophores (log_10_ values, ordinate) are compiled against autofluorescence (orange bars) for every channel (abscissa). The light signal range for each channel is given in the Methods section. Colour-coded distributions of data by concentration of fluorophore (micromolar) are shown. The median values (log_10_) in channel RL1 for DiSC3 and BL3 for pyronin Y are 50-fold (at 1.3 µM) and 4-fold (at 1.3 µM) above autofluorescence, respectively. In channel BL1 SYBR Green I (stock diluted 10000×) and thiazole orange (10 µM) showed log_10_ values above autofluorescence equivalent to 3.2-fold and 4-fold, respectively. The chosen channel for each fluorophore is mapped with a black vertical arrow. Each concentration is represented by eight biological replicates taken over a period of 5 months.

**Fig. 5.**
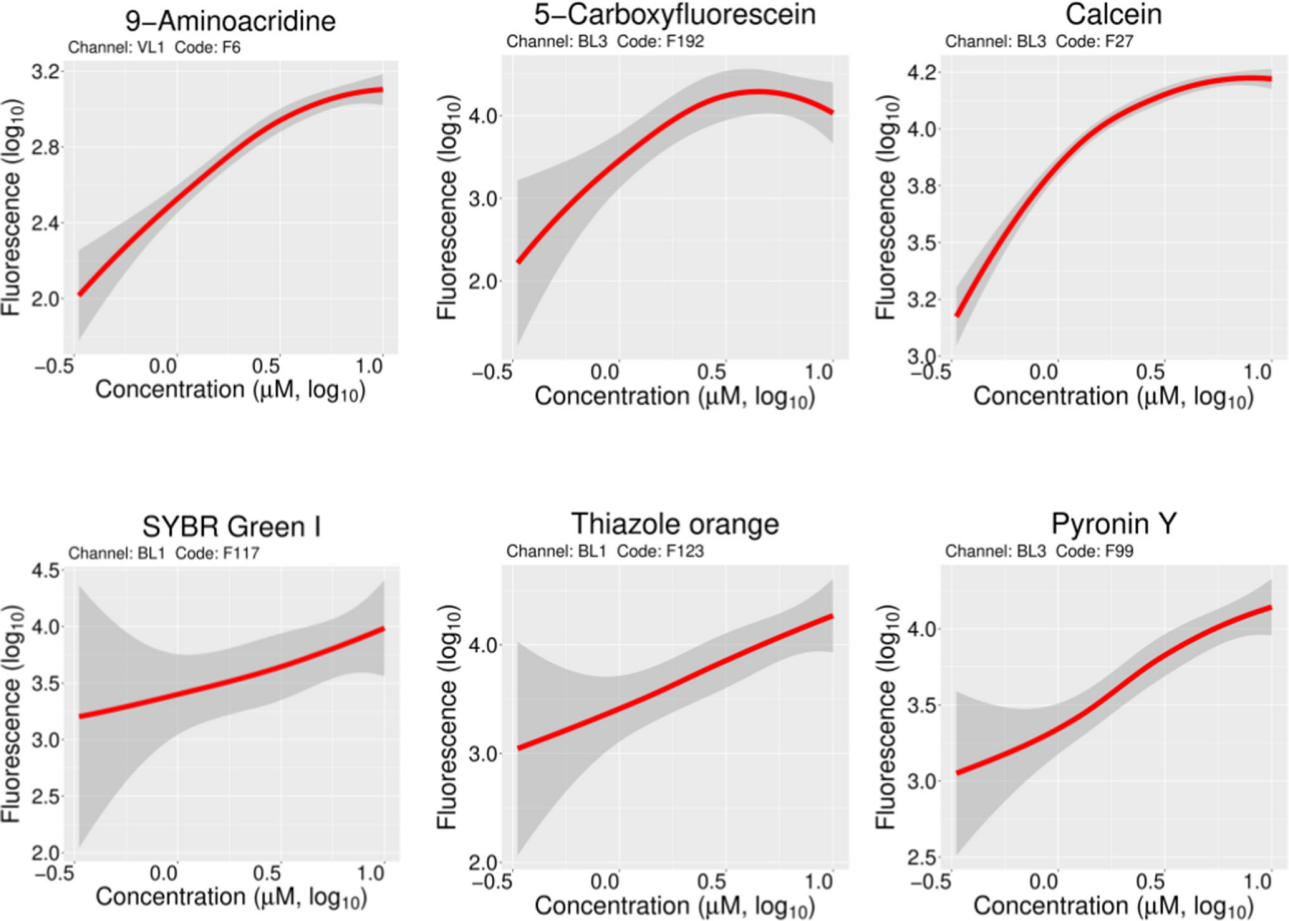
Effect of concentration on fluorophore uptake. Locally weighted scatterplot smoothing was applied to the fluorescence signal versus fluorophore concentration (1, 2.5, 3, 5, 7.5 and 10 µM). These data were from 6 of the 47 initially chosen fluorophores (see also Fig. S2). Three fluorophores detected in channels VL1 (9-aminoacridine) and BL3 (5-carboxyfluorescein and calcein) only showed linearity up to 1 µM. The dose–response trend was essentially linear for SYBR Green I (channel BL1), thiazole orange (channel BL1) and pyronin Y (channel BL3) up to 10 µM.

A set of 47 fluorophores that showed median values at least twofold (log_10_ greater than ~0.3) more than the median of the autofluorescence for a given channel at the concentration used was the core of the set for the next phases of the study (Tables 1 and S1). Inspection of the data showed no particular bias towards either low MW, polarity, or excitation wavelengths. Two of these dyes are the two that we had used previously (DiSC3(5) and SYBR Green I) [19, 20]. The compiled data for four of these molecules (DiSC3(5), pyronin Y, SYBR Green I and thiazole orange) are shown in Fig. 4. They display their strongest signals in channels RL1, BL3, BL1 and BL1, respectively. The set of 47 molecules displayed a reasonable dose response, as judged by accumulated fluorescence (ignoring autofluorescence) in the concentration range 0.1 to 10 µM (Figs 5 and S3).

Eight dyes that were quite expensive or of unknown/unavailable structure (i.e. SYTO-13, Cruz Fluor 405 and the six ATTO fluorophores) from the set of 47, were usually not used in follow-up experimentation (except for the gene knockout strain screening shown later). When evaluating the structural similarity of the resultant 39 molecules (Fig. 1), a number were phenothiazine derivatives: acridine orange, pyronin Y, 9-aminoacridine, azure A, azure B, azure C, methylene blue and thionine. Xanthene dyes [55] include rhodamines, calcein, fluoresceins and eosin.

Fluorescein derivatives were the second largest group: fluorescein, 5-carboxyfluorescein and 2′,7′-dichlorofluorescein (DCF), rhodamine 800, calcein and doxorubicin (an antineoplastic antibacterial) (Fig. 2). The predicted molecular charges (seen as a decisive property for membrane transport) for these fluorophores showed that NBDG, acridine orange and sulphorhodamine B are expected to be zwitterionic at neutral pH (Fig. 2). Six other fluorophores are expected to have a net negative charge and 18 to have a positive charge (cationic dyes) (Fig. 2). An anticipated example of the latter included the nucleic acid-binding fluorophores (i.e. SYBR Green I, thiazole orange, ethidium bromide and pyronin Y), some of which increase their fluorescence considerably upon such binding. It is of interest that a number are natural products, some with significant molecular masses (e.g. sennoside A, MW 862.7; 7-aminoactinomycin D, MW 1270.4, Table S1), consistent with the view [39] that many transporters evolved and were selected to transport natural products. Pyranine and amaranth are trisulphonated dyes (and they cluster), and how they might get into cells is of some interest.

Overall, the dyes cover a heterogeneous swathe of chemical space (an overall median Tanimoto similarity below 0.6), and very few form clusters with a Tanimoto similarity greater than 0.75, the lower end of the cutoff region in typical cheminformatic analyses for similar bioactivities [39, 56–59]. The structural heterogeneity is reasonably equated with functional heterogeneity in uptake via different transporters, and this is assessed next.

### Membrane transport mediates intracellular fluorophore accumulation

**Fig. 6.**
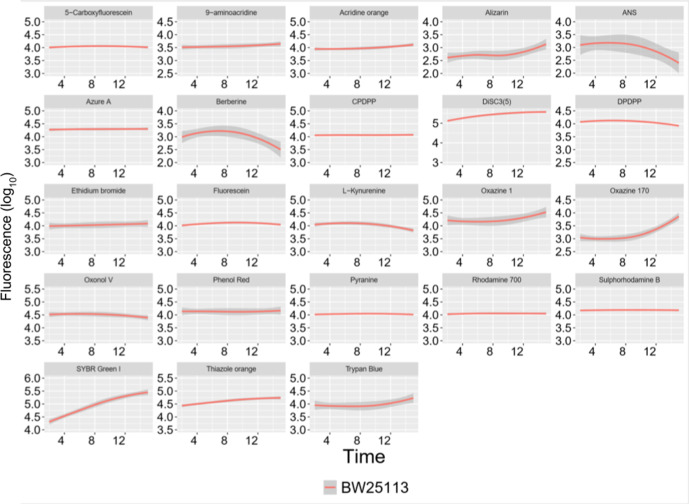
Representative time courses for fluorophore uptake. Locally weighted scatterplot fitting was applied to the fluorescence signal versus time data to follow the levels of fluorophore uptake in the time frame relevant to the experimental settings. *x*-axis: time in minutes (up to 15). *y*-axis: the log_10_ of fluorescence signals. For the fluorescence uptake experiments with different incubation times. The subset shown consisted of 23 dyes out of the 39 dyes used earlier.

Most of the fluorophores of interest were taken up rapidly by *E. coli* BW25113, and the intracellular fluorescence as determined flow cytometrically had reached levels within 2 min that did not vary substantially at 15 min (Fig. 6). A few fluorophores such as DiSC3(5), oxazine 170, SYBR Green I, and thiazole orange were accumulated more slowly, but had reached an approximate steady state by the end of 15 min.

Another important parameter that can affect membrane transport is external pH. The accumulation of all fluorophores tested could vary somewhat with pH in the range tested (pH 6–8.5), with the highest effect (up to two orders of magnitude) seen in the DPP-based dyes CPDPP and DPDPP (Fig. S4). For these two molecules the reduction in their signals is likely due in part to the quenching of their fluorescence because of the deprotonation of the lactam nitrogen in the DPP core [60]. The pH-related behaviour of the other fluorophores is also presented in Fig. S4.

**Fig. 7.**
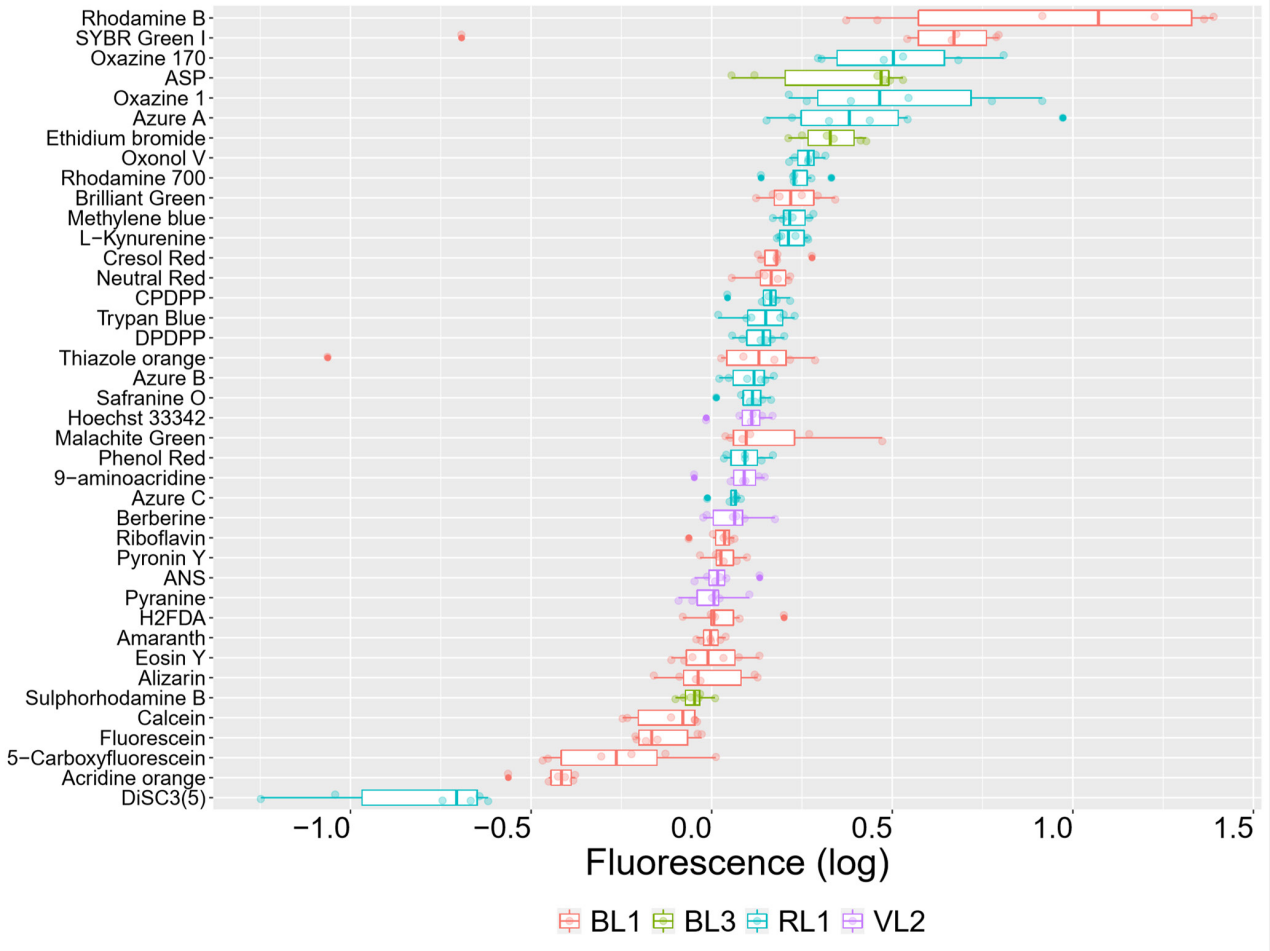
Effect of CPZ in the uptake of fluorophores in LB at pH 8.5. Fluorescence signals are presented as the log_10_ of ratios of the fluorescence from treated cells (* E. coli* BW25113 and 10 µM CPZ) over the fluorescence signals from untreated cells. This time the fluorophore uptake incubation (37 °C, 15 min) was carried out at pH 8.5, which showed the strongest deviations from a ratio of 1 (log_10_=0). Boxplots are ordered by median values. Colours encode the IntelliCyt’s fluorescence channels used.

**Fig. 8.**
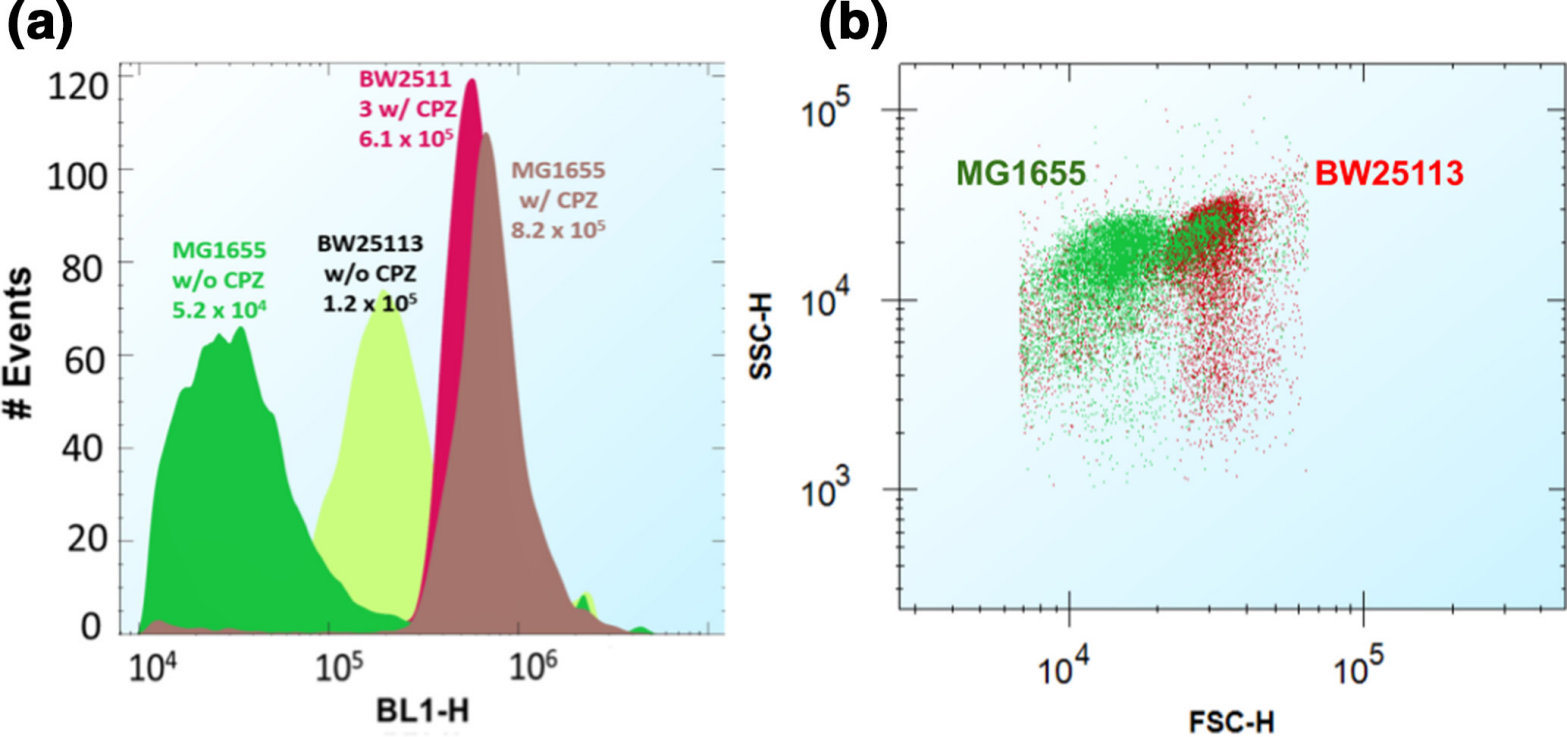
Difference in fluorophore accumulation and light scattering between *E. coli* strains MG1655 and BW25113. (a) Cytograms showing the effects of CPZ on the uptake of SYBR Green into the two strains. The experiments were performed using the same settings published in [20], as also mentioned in the Methods section. (b) Dot plots of light scattering for these two strains. FSC-H, forward scattering; SSC-H, side scattering. Both strains were processed identically and in parallel for these fluorophore uptake assays.

Chlorpromazine is a known efflux inhibitor in *E. coli* [20, 61–63]. In strain BW25113, CPZ increased the accumulation of rhodamine B by an order of magnitude and that of SYBR Green by four- or fivefold (Fig. 7). The fluorescence signal from oxazine 1, oxazine 170 and ASP [4-(4-(dimethylamino)-styryl-N-methylpyridinium (ASP+)] was increased threefold. Here, however, the opposite effect was shown for DiSC3(5), with CPZ inducing a decreased uptake of some threefold. We previously published significantly greater effects of CPZ on SYBR Green I uptake in *E. coli* MG1655 [20] (almost a 20-fold increase). We repeated these experiments for strain MG1655, with the same results as previously. The explanation for the difference observed with strain BW25113 is that BW25113 can accumulate far more di-SC3(5) in the absence of CPZ than does strain MG1655 under the same conditions. The uptake in the presence of CPZ is fairly similar for the two strains, implying a much lowered basal expression of efflux pumps such as acrAB/tolC in strain BW32113 (Fig. 8a). The breadth of the distribution of uptake is consistent with this. Such data illustrate the potential for very substantial variation in the uptake of individual dyes between strains of the same organism. Furthermore, we observed significant differences in the light scattering of these two strains of *E. coli* (Fig. 8b), especially in the forward scattering (which reflects differences in cell size distribution [7, 64]).

### Profiling membrane transporters using fluorophores

**Fig. 9.**
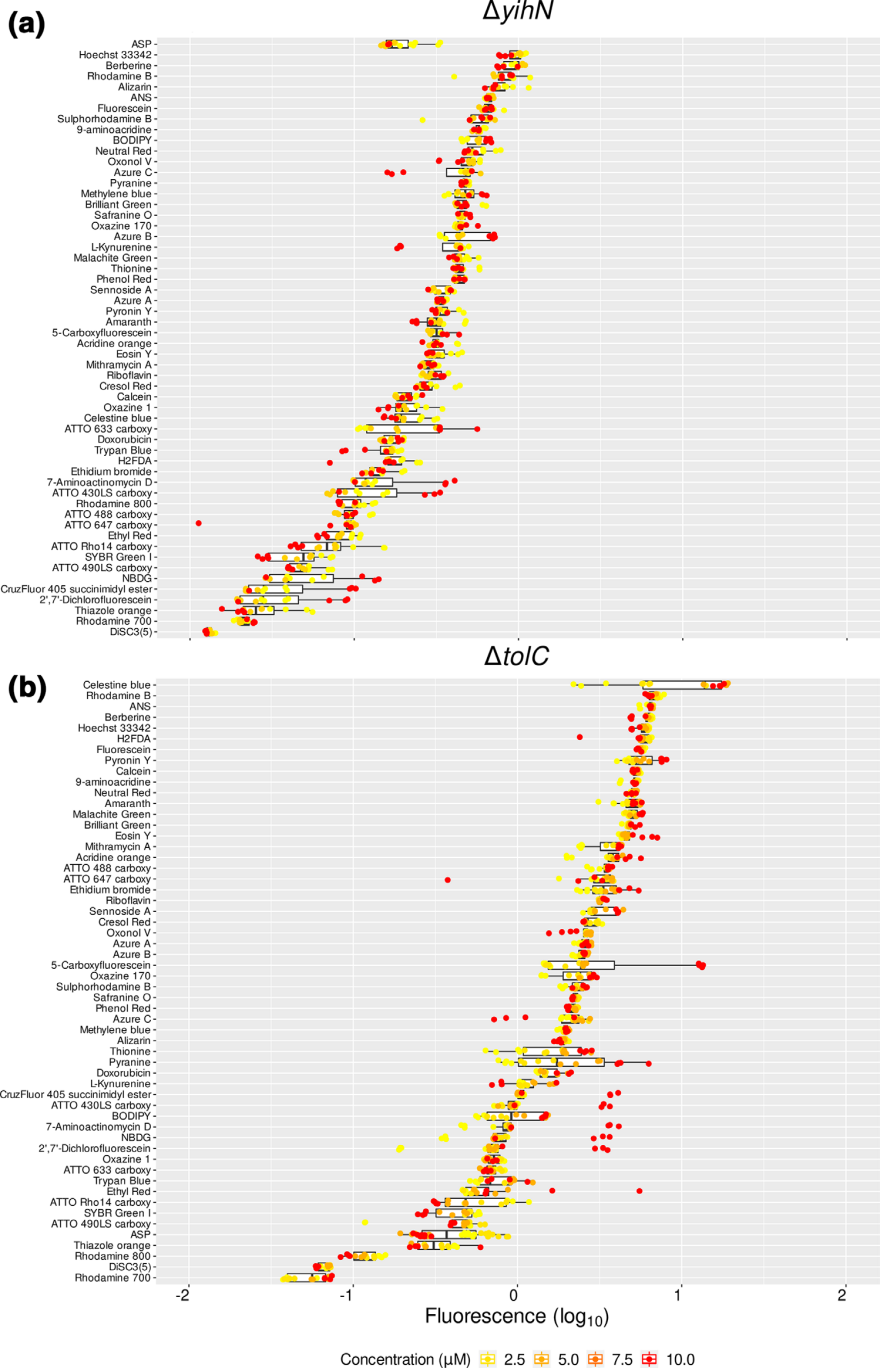
*
E. coli* membrane influxers and effluxers have discriminatory fluorophore accumulation profiles. (a) Fluorophore uptake in the *E. coli* gene knockout strain *yihN*. Fluorescence signals are presented as the log_10_ of ratios of the fluorescence from the *yihN* knockout cells (*∆yihN*) over the fluorescence signals from the reference cells (BW25113). *x*-axis: log_10_ of the ratios (log_10_=0). No difference against the reference strain would have had a ratio of 1 (log_10_=0). (b) Fluorophore uptake in the *E. coli* gene knockout strain *tolC*. Fluorescence signals are presented as the log_10_ of ratios of the fluorescence from the *tolC* knockout cells (*∆tolC*) over the fluorescence signals from the reference cells (BW25113). *x*-axis: log_10_ of the ratios (log_10_=0). No difference against the reference strain would have had a ratio of 1 (log_10_=0). Boxplots are ordered by median (highest on top of the plot). The legend lists the concentrations used for each fluorophore.

As indicated, one of our interests is in the deorphanization of y-gene transporters. An expanded set of fluorophores was used to interrogate the functional traits of strains lacking one of three genes encoding membranes transporters, viz *yhjV*, *yihN* and *tolC*. The success of the gene knockouts was confirmed by PCR (not shown). Two of these genes, *yhjV* and *yihN*, encode transporters with unknown substrates. The third one, *tolC*, encodes an ancillary protein component that is used by a number of different membrane transporters, mainly effluxers. After normalizing for autofluorescence, the y-gene knockouts Δ*yhjV* (Fig. S5) and Δ*yihN* (Fig. 9a) showed a clear ‘influxer’ pattern of fluorescence whereby the median signals of the tested fluorophores were below that of the reference strain (i.e. below a ratio of 1, log_10_=0). For the majority of dyes the uptake responded to concentrations of up to 10 µM. The fluorescence signal ratios of KO strains versus the reference strains are given in Table 2 for all three strains, together with their standard deviations and the 95 % confidence interval.

**Table 2.** Fluorophore uptake profile for three different membrane transporters of *E. coli* BW25113. Fluorescence (Signal Ratios) data are presented as the ratios of the fluorescence (median) from the three different *E. coli* knockout strains (∆*yhjV*, ∆*yihN*, and ∆*tolC*) over the fluorescence (median) from the reference strain (* E. coli* BW25113). No difference between a KO strain and the reference would have had a ratio of 1. SD: standard deviation of the population, CI95: 95% confidence interval. The data for each knockout strain are sorted by incremental signal ratios

yhjV				yihN				tolC			
Fluor	Median	SD	CI95	Fluor	Median	SD	CI95	Fluor	Median	SD	CI95
Thiazole orange	0.29	0.06	[0.26–0.31]	DiSC3(5)	0.01	0	[0.01–0.01]	Rhodamine 700	0.06	0.01	[0.05–0.06]
ATTO Rho14 carboxy	0.37	0.08	[0.34–0.41]	Rhodamine 700	0.02	0	[0.02–0.02]	DiSC3(5)	0.07	0.01	[0.07–0.07]
SYTO13	0.44	0.24	[0.32–0.55]	2',7′-Dichlorofluorescein	0.03	0.03	[0.01–0.04]	Rhodamine 800	0.12	0.02	[0.11–0.13]
SYBR Green I	0.46	0.09	[0.43–0.49]	CruzFluor 405 succinimidyl ester	0.03	0.03	[0.01–0.05]	Thiazole orange	0.31	0.1	[0.26–0.36]
Rhodamine 800	0.55	0.12	[0.49–0.61]	Thiazole orange	0.03	0.01	[0.02–0.03]	DMAMP	0.38	0.2	[0.31–0.45]
Ethyl Red	0.58	0.09	[0.53–0.62]	NBDG	0.04	0.04	[0.02–0.06]	ATTO 490LS carboxy	0.47	0.14	[0.4–0.54]
Sennoside A	0.6	0.07	[0.57–0.64]	ATTO 490LS carboxy	0.05	0.01	[0.04–0.05]	ATTO Rho14 carboxy	0.48	0.28	[0.35–0.62]
Rhodamine B	0.61	0.19	[0.54–0.68]	SYBR Green I	0.05	0.02	[0.04–0.06]	SYBR Green I	0.48	0.12	[0.42–0.54]
Alizarin	0.63	0.06	[0.61–0.65]	ATTO Rho14 carboxy	0.07	0.04	[0.05–0.09]	Ethyl Red	0.55	1.26	[-0.07–1.16]
Amaranth	0.68	0.13	[0.63–0.72]	Ethyl Red	0.08	0.02	[0.07–0.09]	Trypan Blue	0.68	0.22	[0.57–0.79]
Rhodamine 700	0.69	0.17	[0.63–0.75]	ATTO 488 carboxy	0.09	0.02	[0.08–0.1]	ATTO 633 carboxy	0.69	0.07	[0.65–0.72]
Cresol Red	0.74	0.12	[0.7–0.78]	ATTO 647 carboxy	0.09	0.02	[0.08–0.11]	2',7′-Dichlorofluorescein	0.72	1.1	[0.18–1.26]
DiSC3(5)	0.75	0.2	[0.68–0.82]	Rhodamine 800	0.1	0.02	[0.09–0.11]	Oxazine 1	0.72	0.07	[0.69–0.75]
Oxonol V	0.75	0.15	[0.7–0.8]	ATTO 430LS carboxy	0.11	0.1	[0.05–0.16]	NBDG	0.75	1.08	[0.22–1.27]
Phenol Red	0.75	0.23	[0.67–0.83]	7-Aminoactinomycin D	0.12	0.12	[0.05–0.19]	7-Aminoactinomycin D	0.88	1.23	[0.28–1.49]
Malachite Green	0.76	0.17	[0.7–0.82]	Ethidium bromide	0.14	0.03	[0.13–0.16]	BODIPY	0.92	0.39	[0.73–1.11]
Eosin Y	0.77	0.05	[0.75–0.78]	H2FDA	0.16	0.05	[0.14–0.19]	ATTO 430LS carboxy	0.95	1.02	[0.45–1.45]
Sulphorhodamine B	0.77	0.22	[0.7–0.85]	Doxorubicin	0.17	0.02	[0.16–0.18]	CruzFluor 405 succinimidyl ester	1.07	1.12	[0.52–1.62]
Brilliant Green	0.79	0.12	[0.75–0.84]	Trypan Blue	0.17	0.04	[0.15–0.19]	l-Kynurenine	1.08	0.3	[0.94–1.22]
Doxorubicin	0.79	0.12	[0.75–0.84]	ATTO 633 carboxy	0.18	0.14	[0.1–0.26]	Doxorubicin	1.47	0.28	[1.33–1.61]
Trypan Blue	0.79	0.12	[0.74–0.83]	DMAMP	0.18	0.05	[0.16–0.21]	Pyranine	1.74	1.65	[0.94–2.55]
l-Kynurenine	0.8	0.12	[0.75–0.84]	Celestine blue	0.19	0.06	[0.16–0.23]	Thionine	1.89	0.74	[1.53–2.25]
Pyronin Y	0.8	0.17	[0.74–0.85]	Oxazine 1	0.19	0.06	[0.16–0.23]	Alizarin	1.92	0.1	[1.87–1.96]
Celestine blue	0.82	0.05	[0.8–0.85]	Calcein	0.2	0.03	[0.18–0.22]	Methylene blue	1.97	0.09	[1.93–2.01]
ATTO 488 carboxy	0.83	0.18	[0.77–0.9]	Cresol Red	0.26	0.07	[0.23–0.3]	Celestine blue	13.82	6.56	[10.61–17.03]
Thionine	0.84	0.1	[0.81–0.88]	Eosin Y	0.3	0.06	[0.27–0.34]	Azure C	2.2	0.61	[1.9–2.5]
Hoechst 33 342	0.85	0.05	[0.83–0.87]	Mithramycin A	0.3	0.02	[0.28–0.31]	Phenol Red	2.21	0.12	[2.15–2.26]
Neutral Red	0.85	0.09	[0.81–0.88]	Riboflavin	0.3	0.04	[0.28–0.32]	Safranine O	2.27	0.08	[2.23–2.31]
Safranine O	0.85	0.14	[0.79–0.9]	Acridine orange	0.31	0.05	[0.28–0.34]	Sulphorhodamine B	2.29	0.26	[2.17–2.42]
Mithramycin A	0.86	0.18	[0.8–0.92]	5-Carboxyfluorescein	0.32	0.04	[0.29–0.34]	Oxazine 170	2.4	0.59	[2.11–2.69]
9-aminoacridine	0.88	0.06	[0.86–0.9]	Amaranth	0.32	0.08	[0.27–0.36]	5-Carboxyfluorescein	2.44	4.99	[-0.01–4.89]
Azure B	0.88	0.11	[0.84–0.92]	Pyronin Y	0.33	0.05	[0.3–0.36]	Azure B	2.53	0.17	[2.45–2.62]
DMAMP	0.88	0.14	[0.83–0.93]	Azure A	0.34	0.02	[0.33–0.35]	Azure A	2.61	0.18	[2.52–2.7]
ASA	0.9	0.02	[0.89–0.91]	Sennoside A	0.37	0.04	[0.35–0.39]	Oxonol V	2.63	0.35	[2.45–2.8]
ATTO 647 carboxy	0.9	0.17	[0.84–0.95]	Phenol Red	0.41	0.03	[0.4–0.43]	Cresol Red	2.67	0.27	[2.54–2.81]
Berberine	0.9	0.08	[0.87–0.93]	Thionine	0.42	0.06	[0.39–0.46]	Sennoside A	2.93	0.64	[2.62–3.25]
5-Carboxyfluorescein	0.91	0.09	[0.88–0.94]	l-Kynurenine	0.43	0.12	[0.37–0.5]	Riboflavin	3.21	0.12	[3.15–3.27]
ATTO 490LS carboxy	0.91	0.13	[0.85–0.97]	Malachite Green	0.43	0.05	[0.4–0.46]	Ethidium bromide	3.33	0.88	[2.9–3.76]
H2FDA	0.91	0.12	[0.87–0.95]	Azure B	0.44	0.16	[0.35–0.53]	ATTO 647 carboxy	3.44	0.92	[2.99–3.89]
Acridine orange	0.92	0.17	[0.86–0.98]	Oxazine 170	0.44	0.04	[0.42–0.47]	ATTO 488 carboxy	3.56	0.34	[3.39–3.72]
Methylene blue	0.92	0.17	[0.86–0.98]	Safranine O	0.45	0.03	[0.43–0.46]	Acridine orange	3.8	1	[3.31–4.29]
Riboflavin	0.92	0.12	[0.88–0.96]	Brilliant Green	0.46	0.07	[0.42–0.5]	Mithramycin A	4.05	0.75	[3.68–4.42]
Azure A	0.93	0.15	[0.88–0.98]	Methylene blue	0.47	0.1	[0.42–0.53]	Eosin Y	4.5	0.87	[4.07–4.93]
Calcein	0.95	0.13	[0.9–0.99]	Azure C	0.48	0.15	[0.4–0.57]	Brilliant Green	4.78	0.26	[4.65–4.91]
Ethidium bromide	0.95	0.05	[0.93–0.96]	Pyranine	0.48	0.02	[0.47–0.49]	Malachite Green	4.97	0.45	[4.75–5.19]
Fluorescein	0.95	0.1	[0.91–0.98]	Oxonol V	0.51	0.09	[0.47–0.56]	Amaranth	5.02	0.74	[4.66–5.39]
Azure C	0.97	0.04	[0.95–0.98]	Neutral Red	0.52	0.1	[0.47–0.58]	Neutral Red	5.03	0.2	[4.93–5.13]
Oxazine 1	0.98	0.14	[0.93–1.03]	9-aminoacridine	0.57	0.03	[0.56–0.59]	9-aminoacridine	5.18	0.43	[4.97–5.4]
Oxazine 170	0.99	0.15	[0.94–1.04]	BODIPY	0.57	0.08	[0.53–0.62]	Calcein	5.26	0.2	[5.16–5.36]
ATTO 633 carboxy	1	0.17	[0.92–1.09]	Sulphorhodamine B	0.6	0.12	[0.53–0.67]	Pyronin Y	5.27	1.27	[4.65–5.9]
CPDPP	1.01	0.04	[0.99–1.03]	ASA	0.66	0.03	[0.64–0.67]	Fluorescein	5.62	0.2	[5.52–5.72]
Pyranine	1.01	0.15	[0.96–1.07]	Fluorescein	0.66	0.06	[0.62–0.69]	H2FDA	5.86	0.97	[5.39–6.34]
DPDPP	1.04	0.2	[0.94–1.14]	Alizarin	0.73	0.14	[0.65–0.81]	Hoechst 33 342	6.1	0.51	[5.85–6.35]
BODIPY	1.19	0.05	[1.17–1.21]	Rhodamine B	0.79	0.18	[0.69–0.9]	Berberine	6.36	0.61	[6.06–6.66]
				Berberine	0.96	0.12	[0.89–1.03]	ASA	6.51	0.42	[6.3–6.71]
				Hoechst 33 342	1.01	0.12	[0.94–1.08]	Rhodamine B	6.8	0.49	[6.56–7.04]

All fluorophores had a reduced uptake in Δ*yihN*, with most of them accumulating less than threefold the amount in comparison to *E. coli* BW25113 (Fig. 9a). Both rhodamine 700 and DiSC3(5) showed the lowest uptake for any of the concentrations tested (up to 3 µM). Thiazole orange and SYBR Green I were also poorly accumulated by Δ*yihN*, with a more than 10-fold difference relative to the reference strain (Fig. 9a, Table 2).

*E. coli* contains a great many ‘efflux’ pumps, mutations that can cause substantial resistance to multiple drugs and antibiotics (e.g. [65–71]). Many are driven by ATP hydrolysis [72], although others obtain free energy via electron transport-linked membrane energization [73]. A variety of antiporters can also, under some circumstances, appear to act as ‘effluxers’ [32, 74, 75]. TolC is an outer membrane component that interacts with many inner membrane-located transporters (exemplified by acrAB) [67, 76–81]. The involvement of TolC with efflux systems was evident in the pattern of fluorophore uptake observed for 56 dyes in this knockout strain (Fig. 9b, Table 2). Most fluorophores were over-accumulated (ratios over 1, or log_10_ >0). Five fluorophores over accumulated mainly at the higher concentration of 10 µM: fluorescent glucose (NBDG), 2′,7′-dichlorofluorescein, 7-aminoactinomycin D, ATTO 430LS and CruzFluor 450. On the other hand, nine fluorophores had a decreased accumulation of 2-fold or lower in Δ*tolC*, with 10-fold or lower differences in the cases of rhodamine 800, DiSC3(5) and rhodamine 700 (Fig. 9b, Table 2). That the lack of *tolC* seems to impair the transport of the latter dyes points at the interaction or involvement of this protein with outer membrane influx systems (e.g. porins), either directly or via pleiotropic effects [82, 83]. The data for the three knockout strains and the 39 dyes are shown in Fig. S6 (with the six major dyes for the Δ*yihN* knockout being labelled, and with all the data being given in Table 2).

**Fig. 10.**
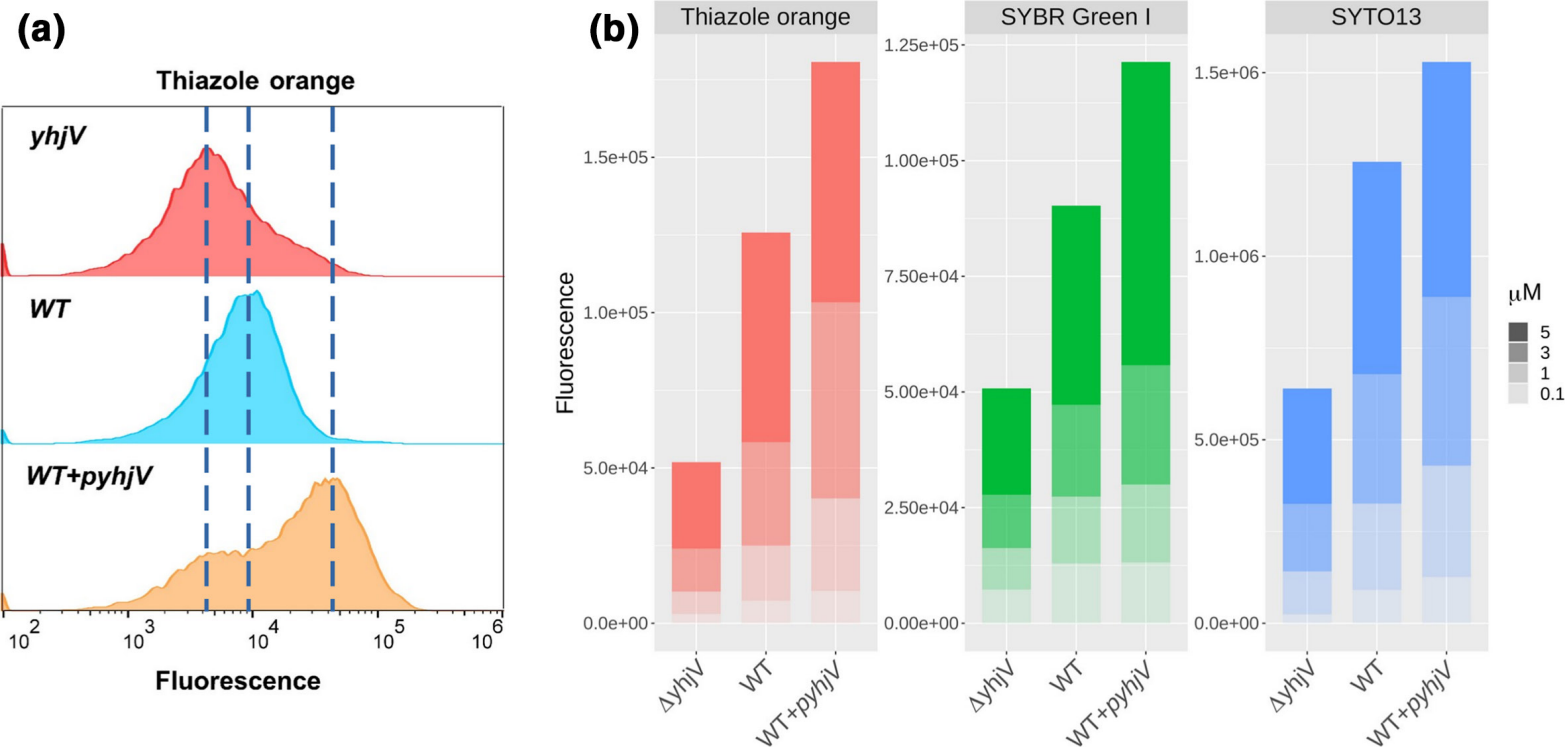
Fluorophores accumulate differentially in *E. coli* overexpressing the YhjV transporter. (a) Cytograms of the uptake of thiazole orange by *E. coli* BW25113 with differing expression levels of *yhjV*. The cell population distribution for the accumulation of thiazole orange (1 µM, 37 °C, 15 min) is presented for the gene knockout of *yhjV* (*∆yhjV*) of the Keio collection. The fluorescence signal of reference strain *E. coli* BW25113 (*WT*) is approximately 2.5-fold higher than the signal from the gene knockout strain (*∆yhjV*). The strain of *E. coli* expressing the *yhjV* gene from the ASKA collection (*WT +pyhjV*) shows a further increase in the accumulation of thiazole orange equivalent to approximately 4.5-fold over the signal from the WT (data taken from the assay where the highest differences were observed for 1 µM). (b) *E. coli* BW25113 overexpressing *yhjV* displays increased uptake of thiazole orange, SYBR Green I and SYTO13. The fluorescence signals of cells accumulating these fluorophores (37 °C, 15 min) are presented for the gene knockout for *yhjV* (*∆yhjV*), the reference strain *E. coli* BW25113 (*WT*) and the strain overexpressing the ASKA construct of *yhjV* (WT +*pyhjV*). Cells were exposed to four different concentrations of fluorophores up to 5 µM, as indicated. Fluorescence data are the averages of the median values from four biological replicates. The accumulation of these fluorophores in all three strains increased monotonically with ascending dye concentrations. For each fluorophore WT cells gave higher fluorescence signals than strains deleted in yhjV, and likewise cells expressing episomal yhjV (WT +pyhjV) over the reference strain (WT). The latter relation for thiazole orange at 1 µM is somewhat smaller (approximately 2.62-fold) than that shown in (a) (4.5-fold), since here we have the average of four experimental replicates.

Finally, we also considered the opposite kind of experiment, in which specific transporter genes are carefully overexpressed, using the equivalent ASKA overexpression clones [84, 85]. Fig. 10a shows the cytograms for the uptake of thiazole orange into the deletant, wild-type and overexpression strains for yhjV when the concentration of the dye was 1 µM, while Fig. 10b shows more extensive data for three dyes at four concentrations. Each of these dyes is clearly capable of providing a surrogate transport assay for the YhjV transporter.

## Discussion

### Role of fluorescent stains in understanding microbial physiology

Fluorescent molecules continue to be instrumental in the versatile, sensitive and quantitative study of microbial structure and physiology. However, the use of fluorophores in the characterization of membrane transporters continues to be sparse (it is done mainly in mammalian cell lines) and is largely directed to the study of drug resistance mediated by efflux pumps (e.g. [36, 86, 87]). Indeed, many drugs show structural similarities with fluorophores [88]. Nonetheless, early evidence has shown that fluorescent dyes such as rhodamine derivatives used in the study of drug efflux require mediated membrane transport influx in the first place [89], and the same is true for nucleic acid stains such as ethidium bromide [90, 91].

Previously, we only studied diSC3(5) [92, 93], which is subject to quenching at high concentrations [94], and SYBR Green I [9, 95–101], where quenching is intramolecular and is stopped (and fluorescence massively enhanced) by binding mainly to dsDNA [102]. Here we have surveyed a large set of fluorophores to assess their suitability as probes for the study of mediated membrane transport in bacteria, initially using the reference strain BW25113 of the Keio *E. coli* gene knockout collection [21].

Regarding the desirable properties of such dyes, as listed in the introduction, we note two caveats: (i) the excitations of those dyes surveyed somewhat reflected the wavelengths of the lasers that we used, and (ii) the eventual choice was largely based on the properties of the wild-type strain. Thus, other dyes might have been included if other excitation wavelengths were available, and/or if other strains showing lower expression of (or lacking) particular efflux pumps had been used.

Out of 143 fluorophores surveyed, the median values of 47 provided signals significantly above autofluorescence to consider pursuing. If those with mean values just 2-fold above autofluorescence are included, this set can be larger (60 fluorophores). However, the latter group of fluorophores are mainly accumulated by the population of higher uptake cells in the right-skewed distributions of fluorescence (Fig. S2c). The accumulation of a core of 39 fluorophores was of particular interest, and these were further characterized after selecting against those of higher cost or with unknown structures. These dyes stained the wild-type BW25113 *E. coli* on short time scales (Fig. 6) in a manner that was suitably dependent on substrate concentration (Figs 5 and S3). Their signals were mostly sensitive to membrane efflux transport inhibitors such as chlorpromazine (Fig. 7), and in some cases to the pH of incubation (Fig. S4).

### Cheminformatics of chosen fluorophores

Despite the reasonable number of available fluorophores, many are not particularly cheap, and our survey was largely confined to the more inexpensive ones available commercially. Cheminformatics [52–54] (sometimes called chemoinformatics [52, 103–107]) describes the discipline that helps researchers assess questions such as the degrees of similarity between individual molecules [108–110] or the molecular diversity within a chemical library [111, 112]. We applied standard cheminformatics methods [39, 50, 113, 114] to the analysis of the relative diversity of our palette of 39 dyes. Given that a pairwise Tanimoto similarity below 0.8 (or a Tanimoto difference exceeding 0.2) is usually taken to mean a significant difference in bioactivity [39, 56–59], it is encouraging that while there were some small clusters (Figs 1 and 2), the median Tanimoto similarity was just 0.6, implying strong orthogonality in the behaviour of our palette, as was borne out experimentally.

This said, many of the diverse dyes were still either phenothiazines or xanthene family dyes, and one conclusion is that the need for a much greater variety of fluorescent scaffolds remains, in order to broaden the present collection yet further.

### Strain differences

It is well known that strain differences between even non-pathogenic *E. coli* can have massive effects even on simple traits such as recombinant protein production [115]. In the present work, in some cases, we observed quite striking differences between the uptake of particular dyes into the two wild-type strains BW25113 and MG1655. In particular, the observed differences in uptake of SYBR Green I in *E. coli* MG1655 and BW25113 might be due to their known genomic differences: *E. coli* BW25113 is Δ(*araD-araB)567* Δ(*rhaD-rhaB)568 ΔlacZ4787* (::rrnB-3) *hsdR514 rph-1*, with the deletion of *araBAD* and *rhaDAB* and the replacement of a section of *lacZ* with four tandem *rrnB* terminators as well as a frameshift mutation in *hsdR* resulting in a premature translation stop codon. *E. coli* BW25113 also contains the *lacI+* allele and not *lacIq*. Other known differences found from genome sequencing are the presence of the *rph-1* allele as well as 20 substitutions and 11 indels [116]. Some of those substitutions directly affect membrane transport systems: *nagE* (N-acetyl glucosamine specific PTS enzyme IIC, IIB and IIA components), *gatC* (subunit of galactitol PTS permease), *btuB* (vitamin B12/cobalamin outer membrane transporter). Furthermore, any single or combined genomic difference can have a number of pleiotropic cellular effects that could easily account for broad phenotypic differences between these two strains of *E. coli* [117, 118]. This is a typical issue of complex systems biology that can cause difficulties yet also has advantages: the difficulties can occur because of an ostensible non-reproducibility of experiments that are in fact different (but in unknown ways), while the advantage is that it shows that our palette of dyes is a particularly strong discriminator of the physiology of different, and even closely related strains. Having previously seen that even single-gene knockouts could induce massive and completely uncorrelated effects in the uptake of particular dyes [20], we sought to assess the utility of this phenomenon in analysing the general dye uptake properties of three membrane transporters, viz YhjV, YihN and TolC.

### The profiles observed with gene knockouts discriminate influx from efflux systems

It is reasonable that the genetic knockout of an import transporter (including antiporters [32, 75]) will tend to result in lower uptake of members of the palette than knockouts of predominantly efflux transporters, that may be expected to have the opposite effect. We assessed this expectation using three transporters, with the expected general results.

As an orphan transporter (marked at Uniprot https://www.uniprot.org/uniprot/P37660 as ‘inner membrane transport protein YhjV’, and as a possible ‘amino acid transmembrane transport protein’), we decided to use YhjV as a first test case for exploiting our dyes. It was chosen because in our previous work [20] the KO strain for *yhjV* accumulated one of the lowest amounts of SYBR Green. There is next to no literature on it, however [119]. Although its main substrates are not known, YhjV is considered to be an uncharacterized member of the hydroxy/aromatic amino acid permease (HAAAP) family within the amino acid/polyamine/organocation (APC) superfamily [120, 121]. To assess other dyes, we compared our palette in terms of uptake between the wild-type and Δ*yhjV* strains (Fig. S5). With the exception of BODIPY, all dyes were taken up less in the knockout than in the wild-type, although only a few by less than threefold. Thiazole orange was an even better (more discriminatory) substrate than SYBR Green. Despite their rather different names, these two dyes are reasonably similar structurally (Fig. 6 and 11), with both including a benzothiazole moiety linked to dual-ring systems. This similarity in behaviour is consistent with the principle of molecular similarity [108], by which similar molecular structures are expected to possess similar bioactivities.

The product encoded by *yihN* is another membrane transporter (https://www.uniprot.org/uniprot/P32135) of unknown function [122, 123]. Here again, essentially no dyes were taken up more in the knockout than in the wild-type (Fig. 9a), again implying that YihN is mainly an influx transporter. In this case, about a dozen dyes were accumulated less than 10-fold in the knockout relative to accumulation in the wild-type.

TolC is well known to be involved in the efflux of a great many substances because it is linked to a variety of inner-membrane efflux transporters [76, 79, 124–126]. In this case the uptake of the majority of the palette was, as expected, greater than in the wild-type in the *tolC* deletion strain. Rhodamines 700 and 800 are hydrophobic (six-ringed) cations, closely related structurally, and are the most and third-most dyes in terms of lowered uptake in the ΔtolC strain (Fig. 9b).

Consequently, the effects of a knockout of a putative transporters on the palette give a clear indication as to whether it is mainly an influx or an efflux transporter (Fig. 9) (although we note that under the conditions of individual assays we cannot discriminate symporters and antiporters; that requires the use of multiple conditions including the putatively antiported substrates [32]).

The above experiments only included gene knockout strains in comparison to the behaviour of the wild-type strain. Overexpression strains provide an arguably more powerful, and at least complementary, approach to understanding the biology of individual proteins. To this end (Fig. 10a), we compared the uptake of thiazole orange in the deletant and overexpression strains of *yhjV*, finding an approximately ninefold variation in uptake of the dye in the two strains, providing a clear use for it as a surrogate dye in assays for this transporter. The same was true of the other three dyes (Fig. 10b) chosen from the top four in the palette (the ATTO dyes were ignored on grounds of cost).

### Pleiotropy

As with any other system based on specific genetic manipulations (e.g. [127, 128]), changes in the expression profile of an individual gene lead to changes in those of many others, a phenomenon referred to as pleiotropy. If *X* changes *Y* and *Y* changes *Z*, but *Y* is not measured, one might naively infer that *X* causes *Z* directly. Clearly, in our case *X* is a transporter activity and *Z* the extent of uptake of a dye of interest. Of course, one might add that the vast majority of studies designed to infer gene function simply perform the equivalent of varying *X* and only measuring *Z*. However, we have built confidence in our method for identifying true transporter substrates in a variety of ways. For instance, we can vary the concentration of our target gene both up and down; pleiotropic effects are not monotonic, although gene activities largely mirror gene expression levels. Similarly, we recognize that by using a palette of dyes we can observe the ordering of changes in dye uptake contingent on changing the expression of a gene; if (say) the effects of *yihN* were mediated solely by its effects on *tolC*, then their ordering profiles would be identical (and they are not). Ultimately, understanding a transporter’s activity in isolation from others (if that is possible) will rely on methods such as its expression in a system that lacks the activity of interest (e.g. often *Xenopus* oocytes [129, 130]). However, these are very far from being high-throughput methods. That proposed here provides the wherewithal for choosing the transporter of interest for such more detailed studies.

### Concluding remarks

The present results and analysis have, for the first time, provided a reasonably comprehensive set of stains for *E. coli *, and have illustrated how sensitive their uptake can be to genotype and physiology. Clearly, the same strategy can be applied to the phenotyping of other microbes of interest, where different preferred dyes are certain to be found (note that rhodamine 123 [131] and hexidium iodide [132] readily enter intact cells of Gram-positive but not Gram-negative bacteria). The differential uptake of specific dyes in strains knocked out for or overexpressing particular transporters provides a clear means for high-throughput assay of these transporters and libraries of potential variants [133–135]; this will be the subject of future communications.

## Supplementary Data

Supplementary material 1

Supplementary material 2
