# Supplementary material for: A palette of fluorophores that are differentially accumulated by wild-type and mutant strains of Escherichia coli: surrogate ligands for profiling bacterial membrane transporters
Source: Microbiology (Reading). 2021 Jan 6;167(2):001016. doi: 10.1099/mic.0.001016 (PMC8131027; doi:10.1099/mic.0.001016)
Supplement: Supplementary material 1 [file mic-167-016-s001.pdf]

Supplementary Figure 1: *E. coli* BW25113 fluorescence signals for 143 fluorophores. The signals for the non-dye control (Autofluorescence) and all fluorophores (log_{10} values, Y-axis) are compiled for each of the 13 channels of the Intellicyt® flow cytometer (X-axis). The median for autofluorescence (orange bars) is drawn for every channel. Colour coded distribution of data by concentration of fluorophore (micromolar) are shown. The subtitles contain our internal code (Fxxx). The number of biological replicates (***n***) for each fluorophore at a given concentration represent data from different experiments carried out over a period of five months.

Supplementary Figure 2. Compilation of the fluorescence detected for 143 fluorescent molecules by the Intellicyt® in *E. coli* BW25113. A: Density distribution of the fluorescence of the full set of fluorophores (blue dash line) using their individual optimal excitation-emission channels at their optimal concentrations (as in Supplementary Table 1). **B**: The signals for all fluorophores are compiled against autofluorescence for each channel. Colour-coded distribution of data by concentration of fluorophore (micromolar) are shown. Orange bar: median value of autofluorescence (note that some dyes can quench autofluorescence). **C**: Density distribution after autofluorescence data were subtracted. The median fluorescence was reduced (blue dash line moved to the left) as a consequence of the subtraction of the autofluorescence. **D**: New spread of the signals distribution for all fluorophores for the 13 channels after subtracting autofluorescence. Colour-coded distribution of data by concentration of fluorophore (micromolar).

Supplementary Figure 3: Dose-response fluorophore uptake. Regression analysis for each of the initial 47 fluorophores that had a two-fold or higher activity (in terms of median values of uptake) in *E. coli* BW25113. Locally weighted scatterplot smoothing was applied to fluorescence signal versus fluorophore concentration data. X-axis: the log_{10} of the concentrations in micromolar. Y-axis: the log_{10} of fluorescence signals. The subtitles contain the relevant channel for each fluorophore and our internal code (Fxxx).

Supplementary Figure 4: Effect of pH and CPZ on the uptake of 40 fluorophores. *E. coli* BW25113 (10^6 cells.mL^{-1}) were incubated with 3 µM fluorophores for 15 minutes at 37ºC (red lines). Similar samples were incubated with both 3 µM fluorophores and 10µM CPZ under the same experimental conditions (blue lines). The incubations were carried out at different pH values: 6.0, 6.5, 7.0, 7.5, 8.0, and 8.5. X-axis: pH values. Y-axis: log_{10} of fluorescence signals after locally weighted scatterplot smoothing.

Supplementary Figure 5: Effect of *yhjV* deletion on fluorophore uptake in *E. coli* BW25113. Fluorescence signals are presented as the log_{10} of ratios of the fluorescence from the *yhjV* knock out cells (Δ*yhjV*) over the fluorescence signals from the reference cells (BW25113). X-axis: log_{10} of the ratios (log_{10} = 0). No difference between the two strain would have a ratio of 1 (log_{10} = 0). Boxplots are ordered by median (highest on top of the plot). The legend lists the concentrations used for each fluorophore. The legend lists the concentrations used for each fluorophore. The clearly related structures of the two most differentiating dyes from the palette of 39 are shown.

Supplementary Figure 6: Differential uptake of 39 dyes between three different knockout strain of *E. coli*. Experiments were performed as described in the legends to Supplementary Figure 5 and 11 Figure 9, and in Table 2. Several dyes showing the largest effect in Δ*yihN* are marked.

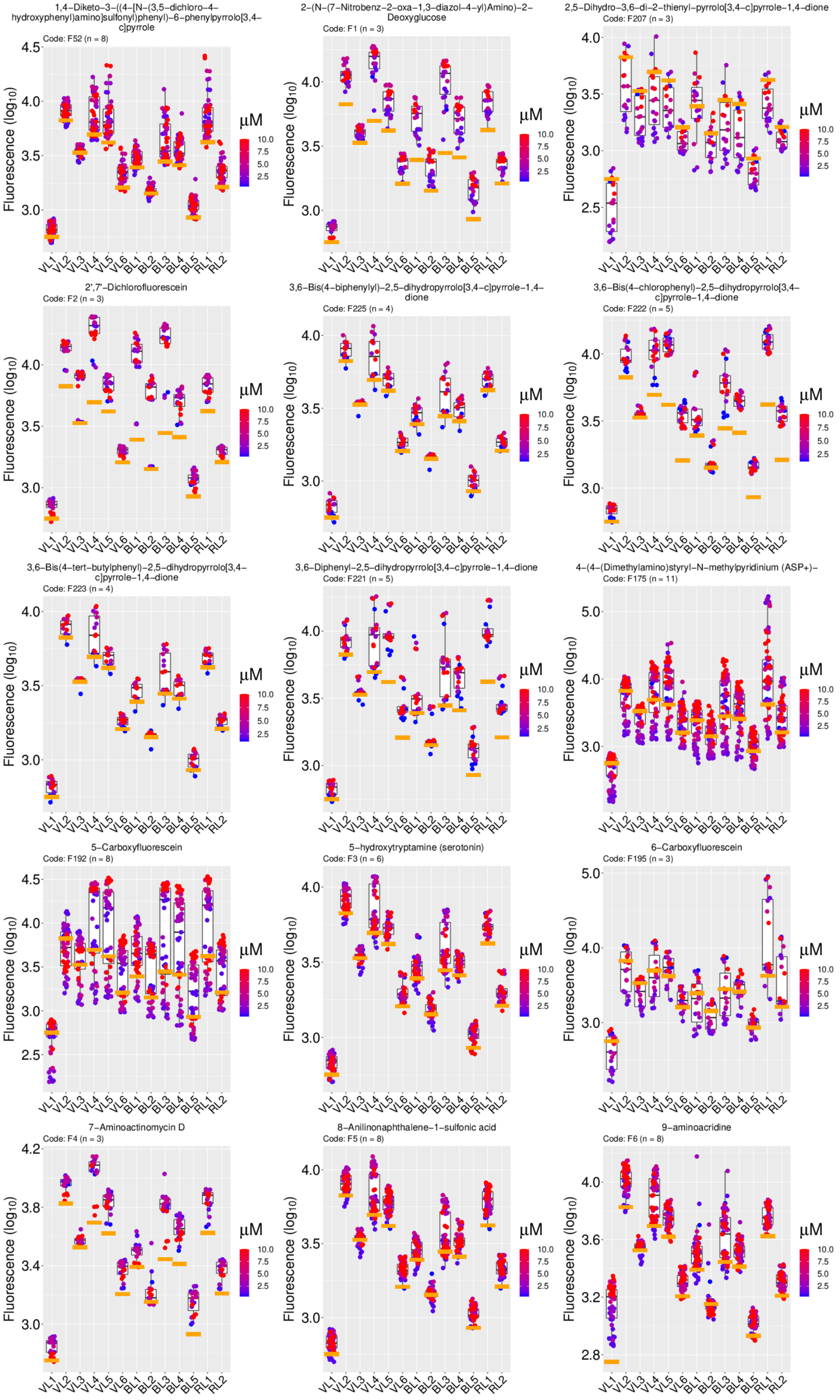
1,4–Diketo–3–((4–[N–(3,5–dichloro–4–hydroxyphenyl)amino]sulfonyl)phenyl)–6–phenylpyrrolo[3,4–c]pyrrole
Code: F52 (n = 8)
2–(N–(7–Nitrobenz–2–oxa–1,3–diazol–4–yl)Amino)–2–Deoxyglucose
Code: F1 (n = 3)
2,5–Dihydro–3,6–di–2–thienyl–pyrrolo[3,4–c]pyrrole–1,4–dione
Code: F207 (n = 3)
2',7'–Dichlorofluorescein
Code: F2 (n = 3)
3,6–Bis(4–biphenylyl)–2,5–dihydropyrrolo[3,4–c]pyrrole–1,4–dione
Code: F225 (n = 4)
3,6–Bis(4–chlorophenyl)–2,5–dihydropyrrolo[3,4–c]pyrrole–1,4–dione
Code: F222 (n = 5)
3,6–Bis(4–tert–butylphenyl)–2,5–dihydropyrrolo[3,4–c]pyrrole–1,4–dione
Code: F223 (n = 4)
3,6–Diphenyl–2,5–dihydropyrrolo[3,4–c]pyrrole–1,4–dione
Code: F221 (n = 5)
4–(4–(Dimethylamino)styryl–N–methylpyridinium (ASP+)–
Code: F175 (n = 11)
5–Carboxyfluorescein
Code: F192 (n = 8)
5–hydroxytryptamine (serotonin)
Code: F3 (n = 6)
6–Carboxyfluorescein
Code: F195 (n = 3)
7–Aminoactinomycin D
Code: F4 (n = 3)
8–Anilinonaphthalene–1–sulfonic acid
Code: F5 (n = 8)
9–aminoacridine
Code: F6 (n = 8)
Fluorescence (log10)
µM
10.0
7.5
5.0
2.5
VL1 VL2 VL3 VL4 VL5 VL6 BL1 BL2 BL3 BL4 BL5 RL1 RL2

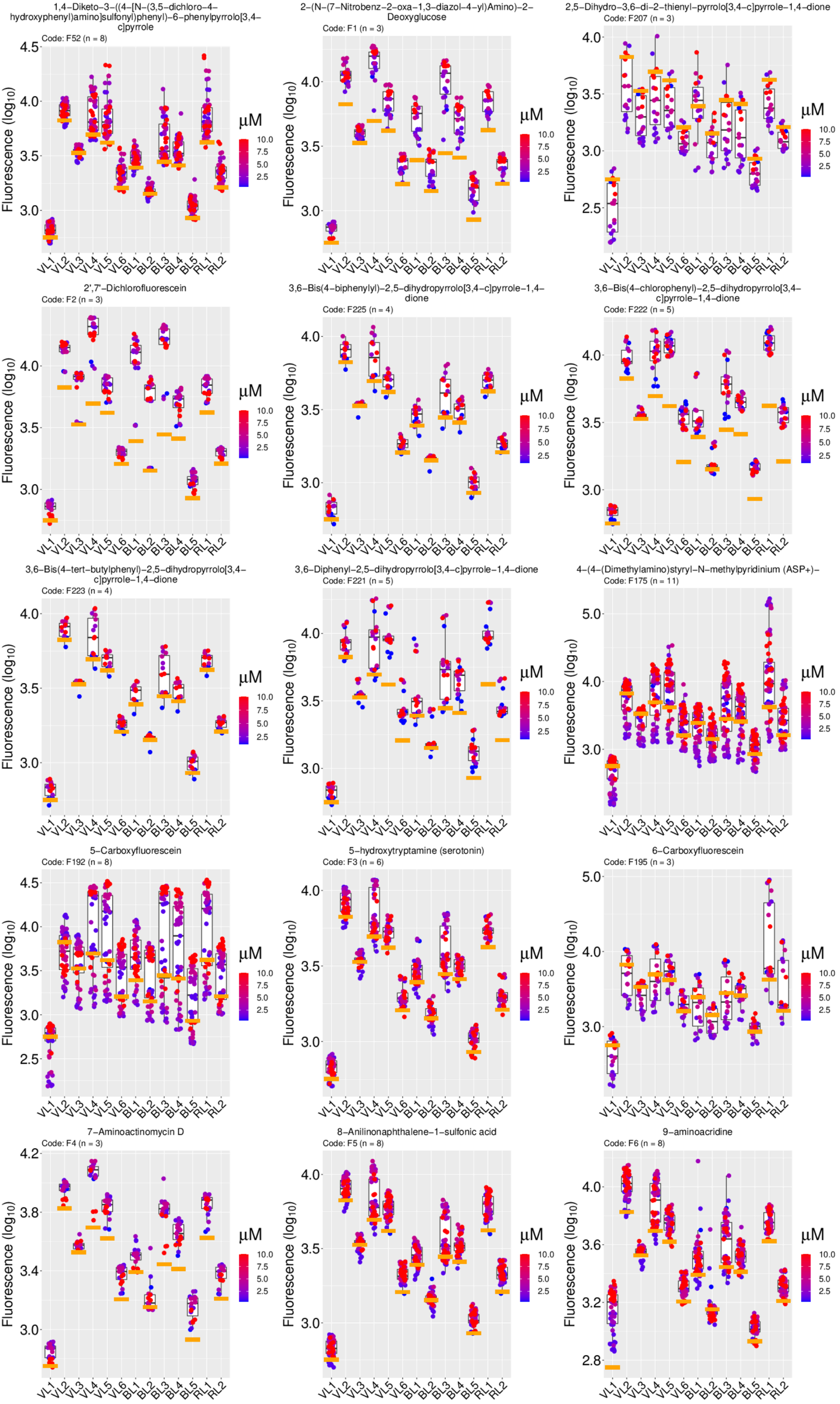
1,4−Diketo−3−((4−[N−(3,5−dichloro−4−hydroxyphenyl)amino]sulfonyl)phenyl)−6−phenylpyrrolo[3,4−c]pyrrole
Code: F52 (n = 8)
2−(N−(7−Nitrobenz−2−oxa−1,3−diazol−4−yl)Amino)−2−Deoxyglucose
Code: F1 (n = 3)
2,5−Dihydro−3,6−di−2−thienyl−pyrrolo[3,4−c]pyrrole−1,4−dione
Code: F207 (n = 3)
2',7'−Dichlorofluorescein
Code: F2 (n = 3)
3,6−Bis(4−biphenylyl)−2,5−dihydropyrrolo[3,4−c]pyrrole−1,4−dione
Code: F225 (n = 4)
3,6−Bis(4−chlorophenyl)−2,5−dihydropyrrolo[3,4−c]pyrrole−1,4−dione
Code: F222 (n = 5)
3,6−Bis(4−tert−butylphenyl)−2,5−dihydropyrrolo[3,4−c]pyrrole−1,4−dione
Code: F223 (n = 4)
3,6−Diphenyl−2,5−dihydropyrrolo[3,4−c]pyrrole−1,4−dione
Code: F221 (n = 5)
4−(4−(Dimethylamino)styryl−N−methylpyridinium (ASP+)−
Code: F175 (n = 11)
5−Carboxyfluorescein
Code: F192 (n = 8)
5−hydroxytryptamine (serotonin)
Code: F3 (n = 6)
6−Carboxyfluorescein
Code: F195 (n = 3)
7−Aminoactinomycin D
Code: F4 (n = 3)
8−Anilinonaphthalene−1−sulfonic acid
Code: F5 (n = 8)
9−aminoacridine
Code: F6 (n = 8)
Fluorescence (log10)
µM
10.0
7.5
5.0
2.5
VL1 VL2 VL3 VL4 VL5 VL6 BL1 BL2 BL3 BL4 BL5 RL1 RL2

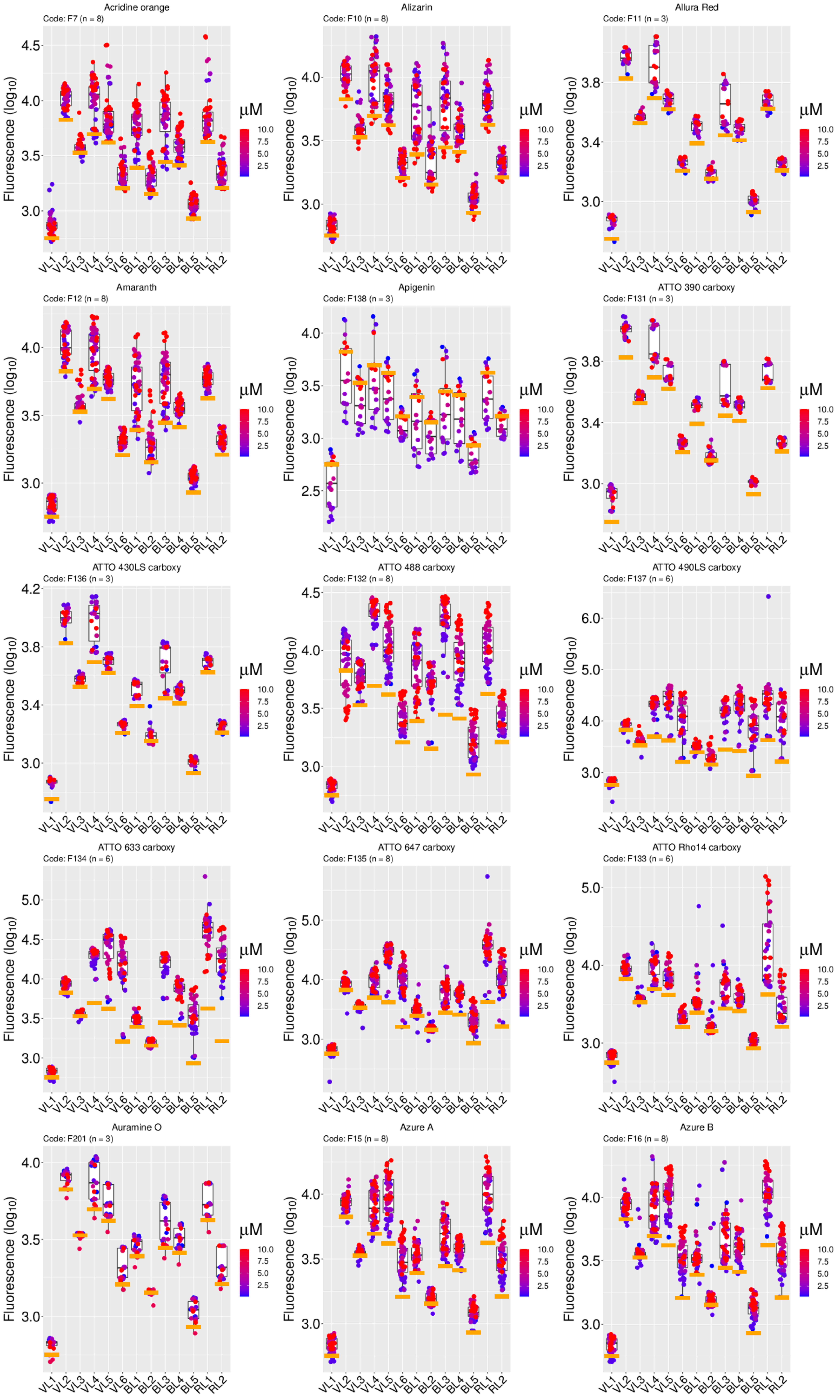
Acridine orange
Code: F7 (n = 8)
Alizarin
Code: F10 (n = 8)
Allura Red
Code: F11 (n = 3)
Amaranth
Code: F12 (n = 8)
Apigenin
Code: F138 (n = 3)
ATTO 390 carboxy
Code: F131 (n = 3)
ATTO 430LS carboxy
Code: F136 (n = 3)
ATTO 488 carboxy
Code: F132 (n = 8)
ATTO 490LS carboxy
Code: F137 (n = 6)
ATTO 633 carboxy
Code: F134 (n = 6)
ATTO 647 carboxy
Code: F135 (n = 8)
ATTO Rho14 carboxy
Code: F133 (n = 6)
Auramine O
Code: F201 (n = 3)
Azure A
Code: F15 (n = 8)
Azure B
Code: F16 (n = 8)
Fluorescence (log10)
µM
10.0
7.5
5.0
2.5
VL1 VL2 VL3 VL4 VL5 VL6 BL1 BL2 BL3 BL4 BL5 RL1 RL2

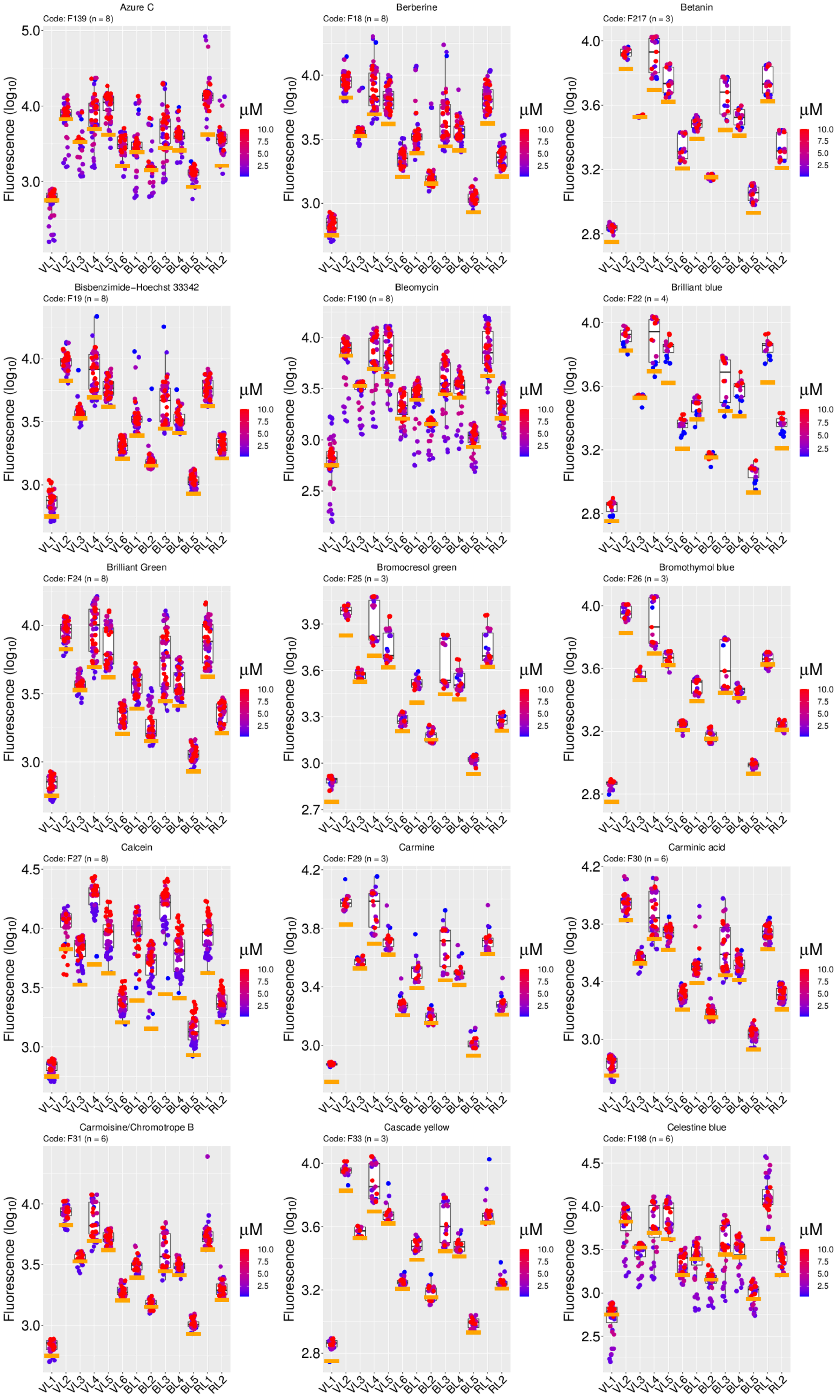
Azure C
Code: F139 (n = 8)
Berberine
Code: F18 (n = 8)
Betanin
Code: F217 (n = 3)
Bisbenzimide−Hoechst 33342
Code: F19 (n = 8)
Bleomycin
Code: F190 (n = 8)
Brilliant blue
Code: F22 (n = 4)
Brilliant Green
Code: F24 (n = 8)
Bromocresol green
Code: F25 (n = 3)
Bromothymol blue
Code: F26 (n = 3)
Calcein
Code: F27 (n = 8)
Carmine
Code: F29 (n = 3)
Carminic acid
Code: F30 (n = 6)
Carmoisine/Chromotrope B
Code: F31 (n = 6)
Cascade yellow
Code: F33 (n = 3)
Celestine blue
Code: F198 (n = 6)
Fluorescence (log10)
µM
10.0
7.5
5.0
2.5
VL1 VL2 VL3 VL4 VL5 VL6 BL1 BL2 BL3 BL4 BL5 RL1 RL2

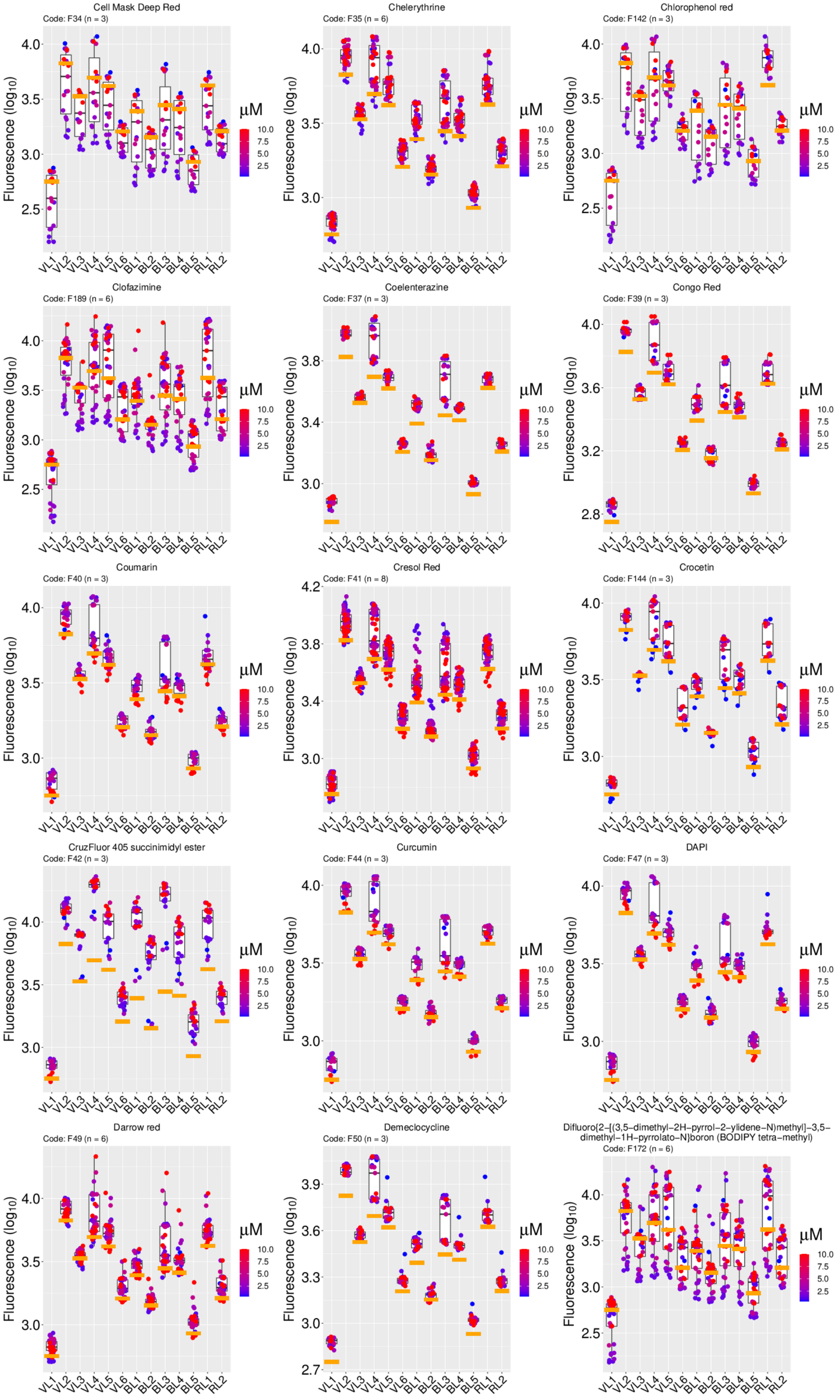
Cell Mask Deep Red
Code: F34 (n = 3)
Chelerythrine
Code: F35 (n = 6)
Chlorophenol red
Code: F142 (n = 3)
Clofazimine
Code: F189 (n = 6)
Coelenterazine
Code: F37 (n = 3)
Congo Red
Code: F39 (n = 3)
Coumarin
Code: F40 (n = 3)
Cresol Red
Code: F41 (n = 8)
Crocetin
Code: F144 (n = 3)
CruzFluor 405 succinimidyl ester
Code: F42 (n = 3)
Curcumin
Code: F44 (n = 3)
DAPI
Code: F47 (n = 3)
Darrow red
Code: F49 (n = 6)
Demeclocycline
Code: F50 (n = 3)
Difluoro{2−[(3,5−dimethyl−2H−pyrrol−2−ylidene−N)methyl]−3,5−dimethyl−1H−pyrrolato−N}boron (BODIPY tetra−methyl)
Code: F172 (n = 6)
Fluorescence (log10)
µM
10.0
7.5
5.0
2.5
VL1
VL2
VL3
VL4
VL5
VL6
BL1
BL2
BL3
BL4
BL5
RL1
RL2

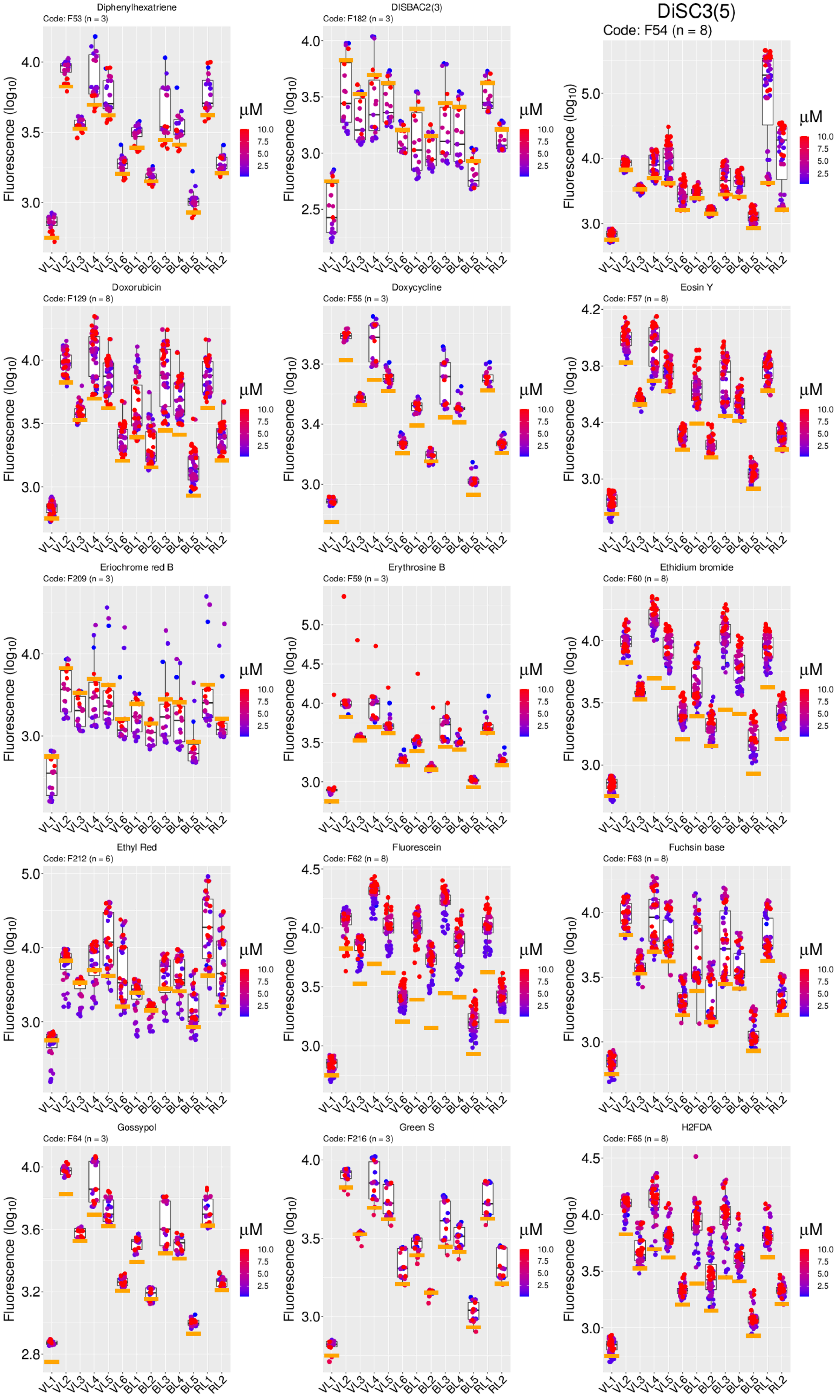

Diphenylhexatriene
Code: F53 (n = 3)
DISBAC2(3)
Code: F182 (n = 3)
DiSC3(5)
Code: F54 (n = 8)
Doxorubicin
Code: F129 (n = 8)
Doxycycline
Code: F55 (n = 3)
Eosin Y
Code: F57 (n = 8)
Eriochrome red B
Code: F209 (n = 3)
Erythrosine B
Code: F59 (n = 3)
Ethidium bromide
Code: F60 (n = 8)
Ethyl Red
Code: F212 (n = 6)
Fluorescein
Code: F62 (n = 8)
Fuchsin base
Code: F63 (n = 8)
Gossypol
Code: F64 (n = 3)
Green S
Code: F216 (n = 3)
H2FDA
Code: F65 (n = 8)
Fluorescence (log10)
μM
10.0
7.5
5.0
2.5
VL1 VL2 VL3 VL4 VL5 VL6 BL1 BL2 BL3 BL4 BL5 RL1 RL2

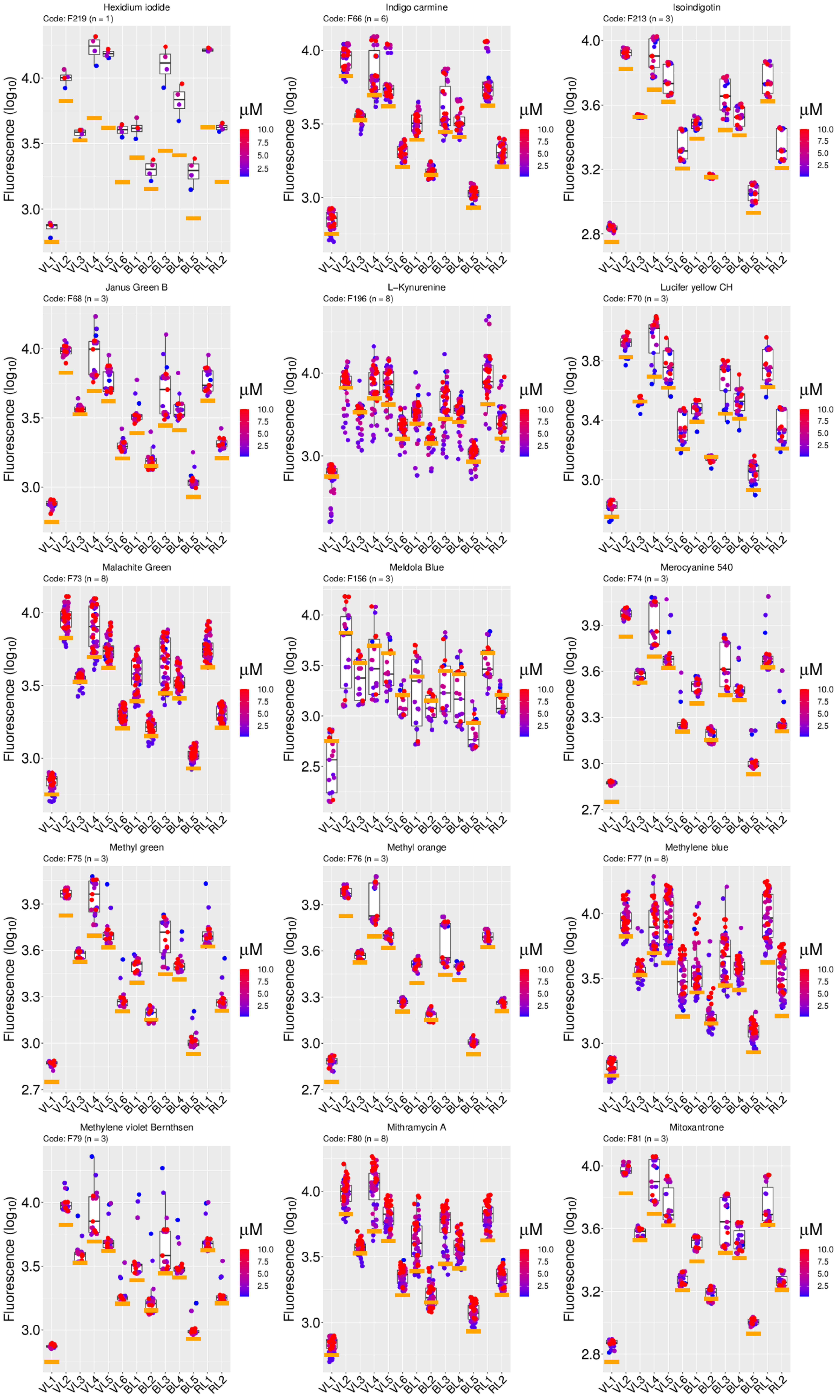
Hexidium iodide
Code: F219 (n = 1)
Indigo carmine
Code: F66 (n = 6)
Isoindigotin
Code: F213 (n = 3)
Janus Green B
Code: F68 (n = 3)
L−Kynurenine
Code: F196 (n = 8)
Lucifer yellow CH
Code: F70 (n = 3)
Malachite Green
Code: F73 (n = 8)
Meldola Blue
Code: F156 (n = 3)
Merocyanine 540
Code: F74 (n = 3)
Methyl green
Code: F75 (n = 3)
Methyl orange
Code: F76 (n = 3)
Methylene blue
Code: F77 (n = 8)
Methylene violet Bernthsen
Code: F79 (n = 3)
Mithramycin A
Code: F80 (n = 8)
Mitoxantrone
Code: F81 (n = 3)
Fluorescence (log10)
µM
10.0
7.5
5.0
2.5
VL1 VL2 VL3 VL4 VL5 VL6 BL1 BL2 BL3 BL4 BL5 RL1 RL2

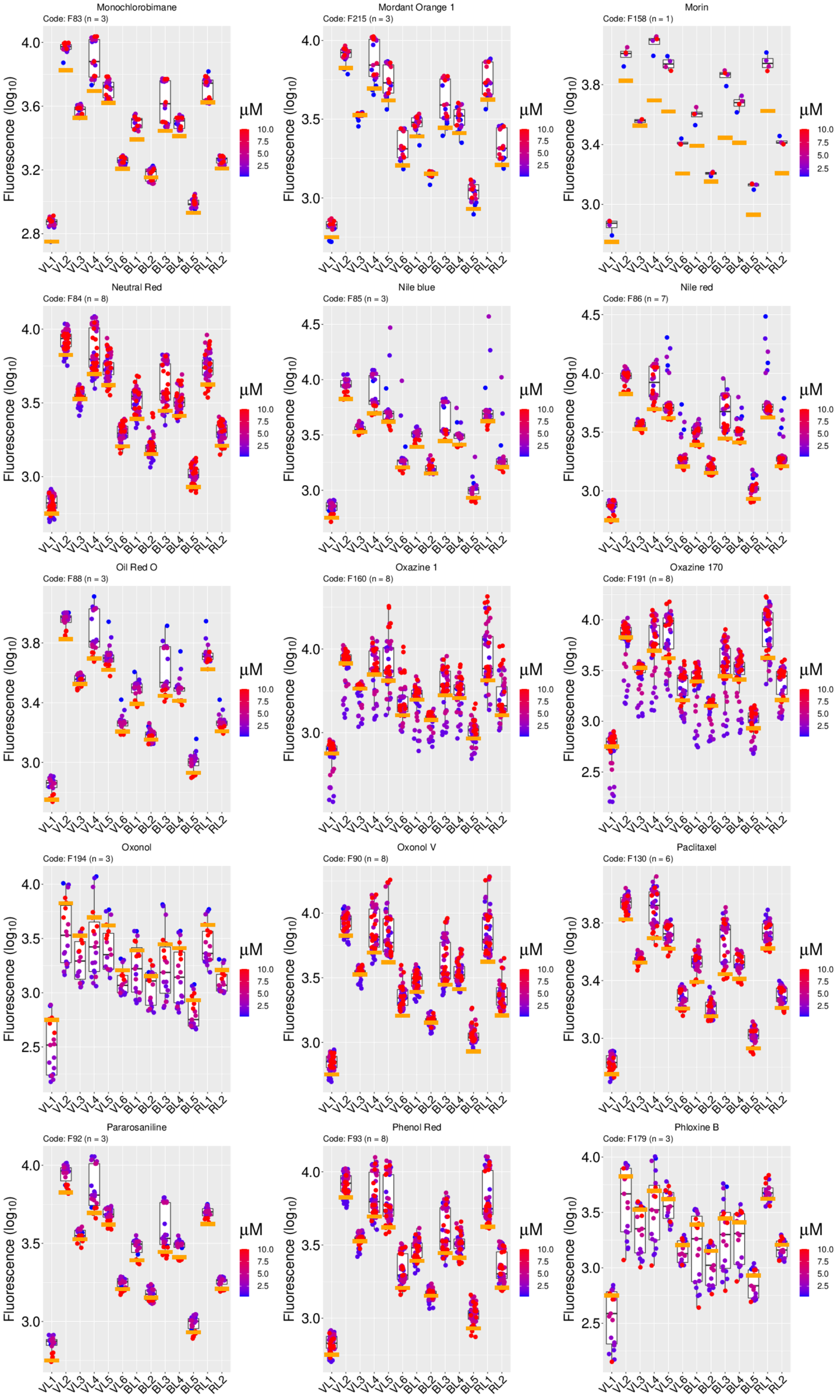
Monochlorobimane
Code: F83 (n = 3)
Mordant Orange 1
Code: F215 (n = 3)
Morin
Code: F158 (n = 1)
Neutral Red
Code: F84 (n = 8)
Nile blue
Code: F85 (n = 3)
Nile red
Code: F86 (n = 7)
Oil Red O
Code: F88 (n = 3)
Oxazine 1
Code: F160 (n = 8)
Oxazine 170
Code: F191 (n = 8)
Oxonol
Code: F194 (n = 3)
Oxonol V
Code: F90 (n = 8)
Paclitaxel
Code: F130 (n = 6)
Pararosaniline
Code: F92 (n = 3)
Phenol Red
Code: F93 (n = 8)
Phloxine B
Code: F179 (n = 3)
Fluorescence (log_{10})
μM
10.0
7.5
5.0
2.5
VL1
VL2
VL3
VL4
VL5
VL6
BL1
BL2
BL3
BL4
BL5
RL1
RL2

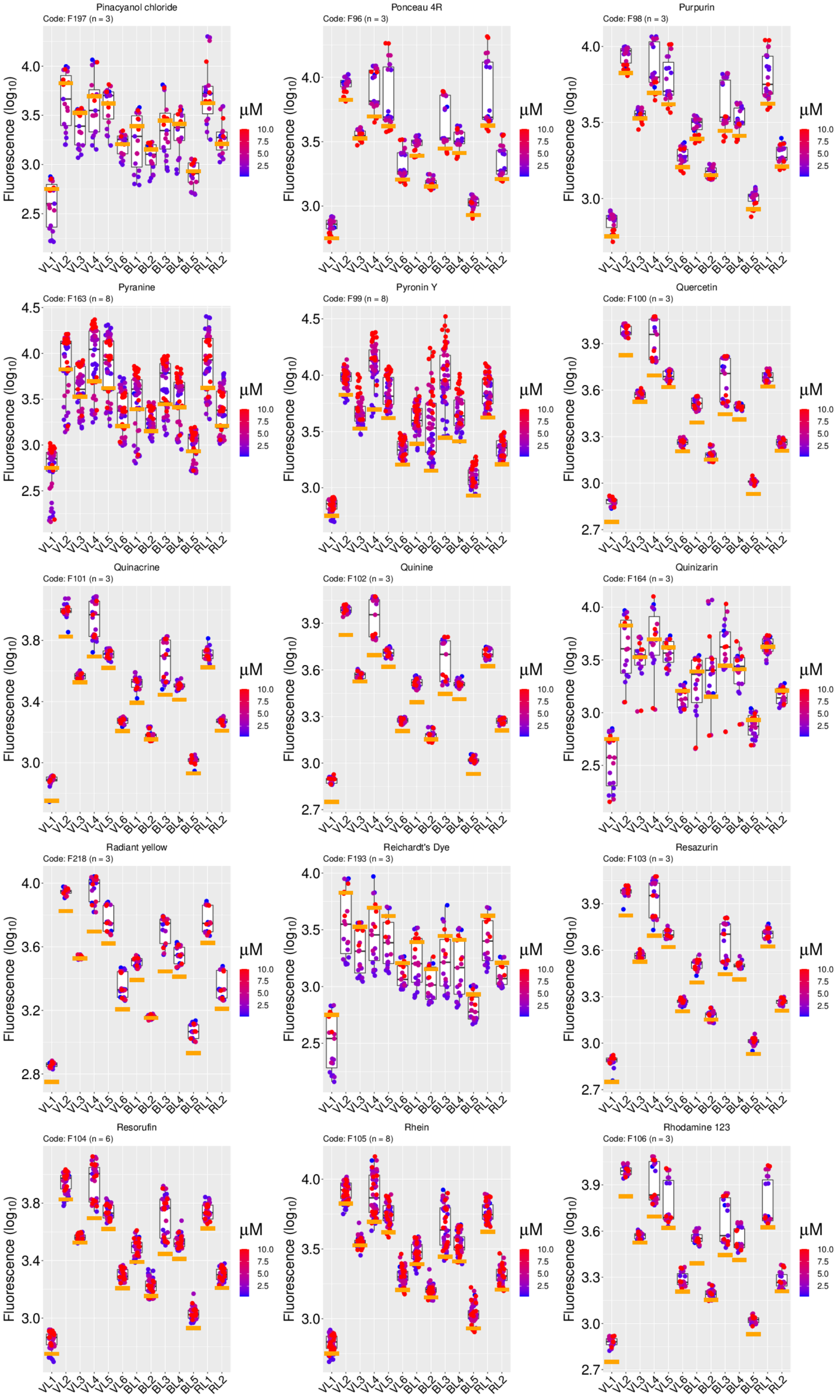
Pinacyanol chloride
Code: F197 (n = 3)
Ponceau 4R
Code: F96 (n = 3)
Purpurin
Code: F98 (n = 3)
Pyranine
Code: F163 (n = 8)
Pyronin Y
Code: F99 (n = 8)
Quercetin
Code: F100 (n = 3)
Quinacrine
Code: F101 (n = 3)
Quinine
Code: F102 (n = 3)
Quinizarin
Code: F164 (n = 3)
Radiant yellow
Code: F218 (n = 3)
Reichardt's Dye
Code: F193 (n = 3)
Resazurin
Code: F103 (n = 3)
Resorufin
Code: F104 (n = 6)
Rhein
Code: F105 (n = 8)
Rhodamine 123
Code: F106 (n = 3)
Fluorescence (log10)
VL1 VL2 VL3 VL4 VL5 VL6 BL1 BL2 BL3 BL4 BL5 RL1 RL2
µM
10.0
7.5
5.0
2.5

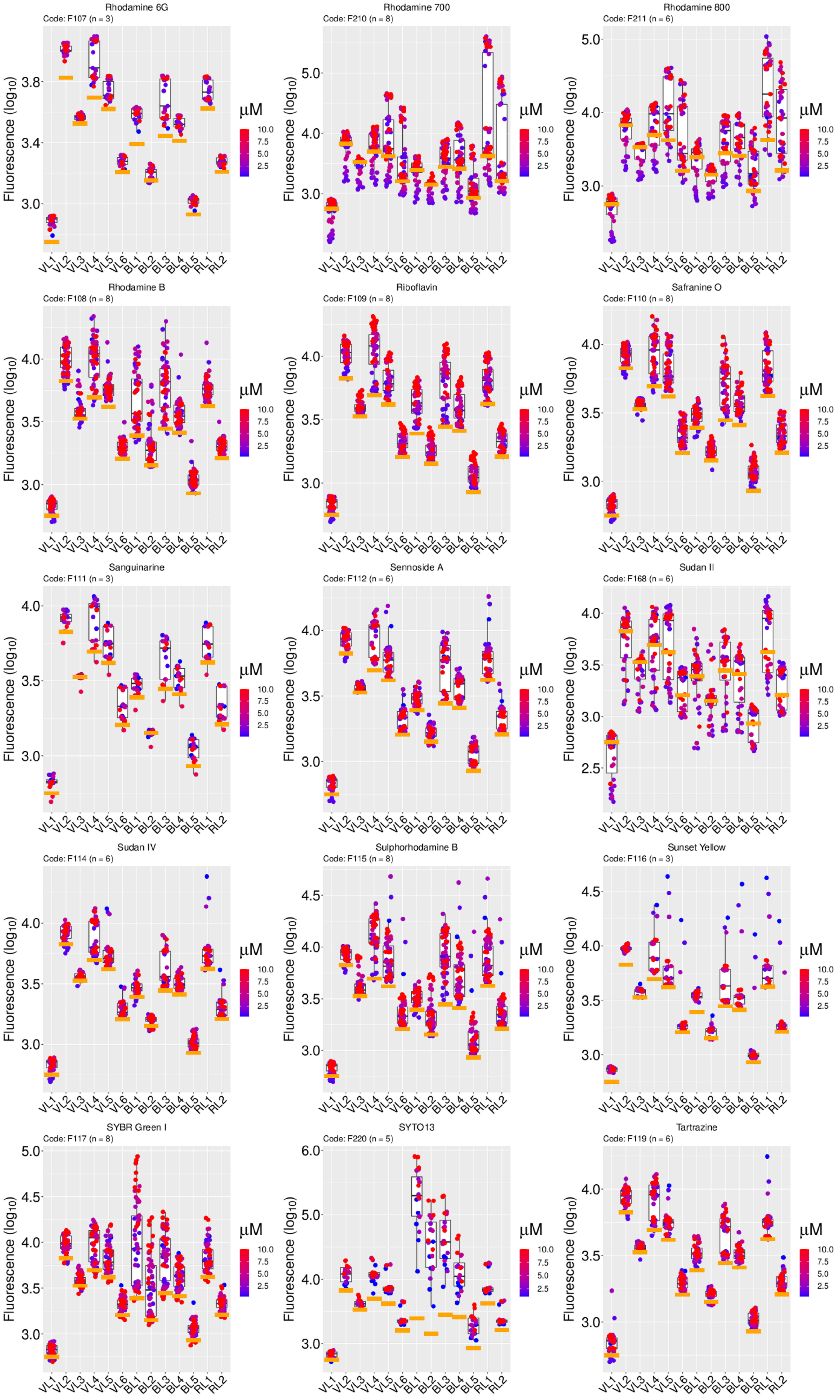

Rhodamine 6G
Code: F107 (n = 3)
Rhodamine 700
Code: F210 (n = 8)
Rhodamine 800
Code: F211 (n = 6)
Rhodamine B
Code: F108 (n = 8)
Riboflavin
Code: F109 (n = 8)
Safranine O
Code: F110 (n = 8)
Sanguinarine
Code: F111 (n = 3)
Sennoside A
Code: F112 (n = 6)
Sudan II
Code: F168 (n = 6)
Sudan IV
Code: F114 (n = 6)
Sulphorhodamine B
Code: F115 (n = 8)
Sunset Yellow
Code: F116 (n = 3)
SYBR Green I
Code: F117 (n = 8)
SYTO13
Code: F220 (n = 5)
Tartrazine
Code: F119 (n = 6)
Fluorescence (log_{10})
μM
10.0
7.5
5.0
2.5
VL1 VL2 VL3 VL4 VL5 VL6 BL1 BL2 BL3 BL4 BL5 RL1 RL2

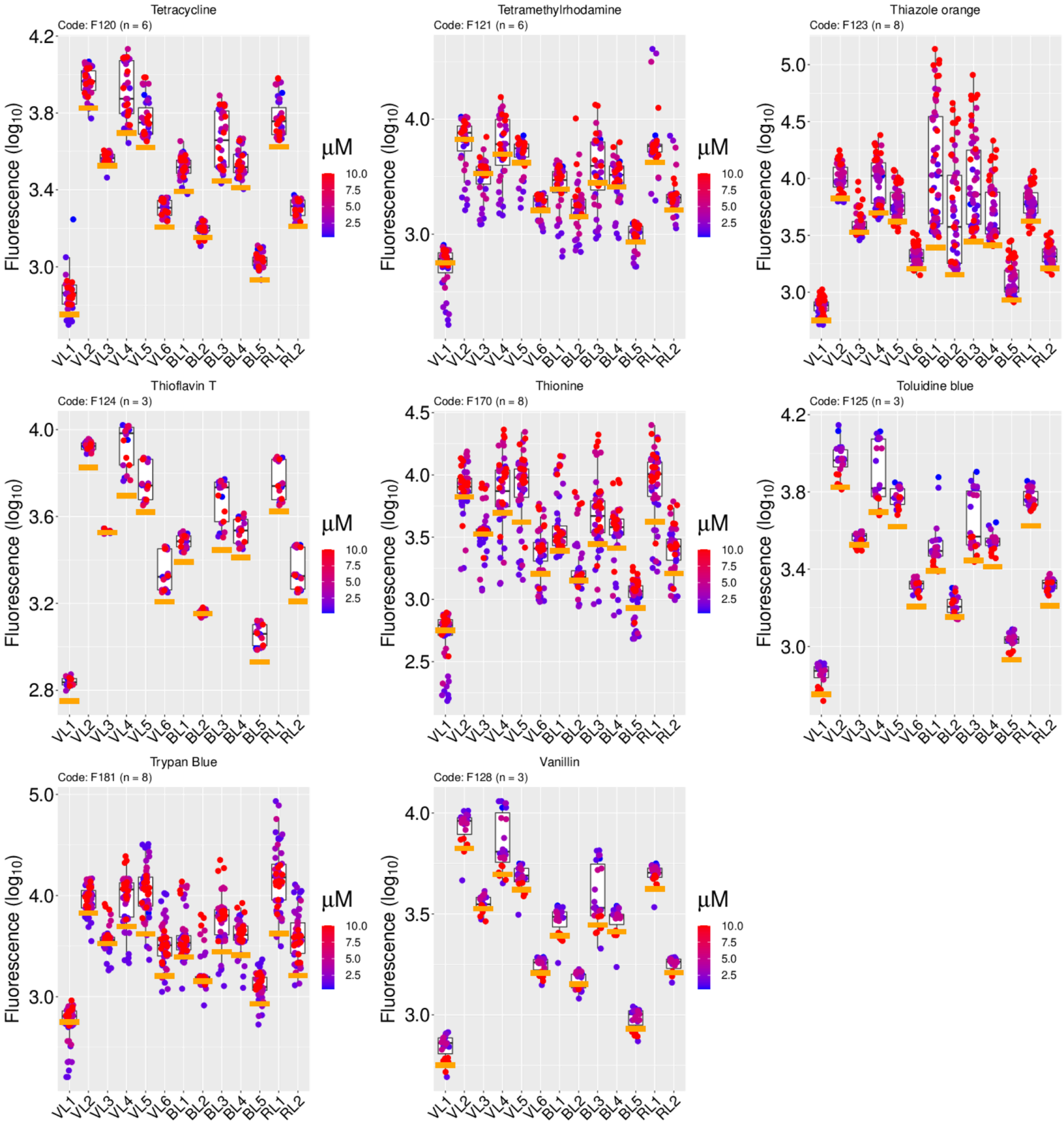

Supplementary Figure 1

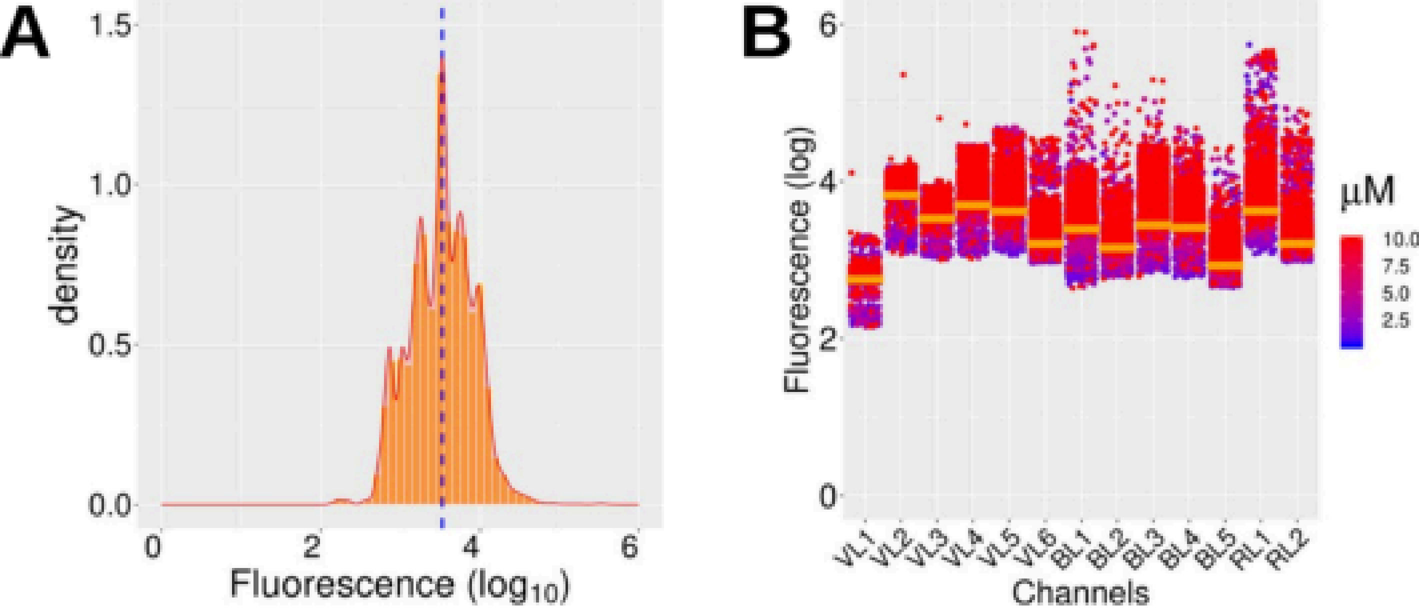

Supplementary Figure 2

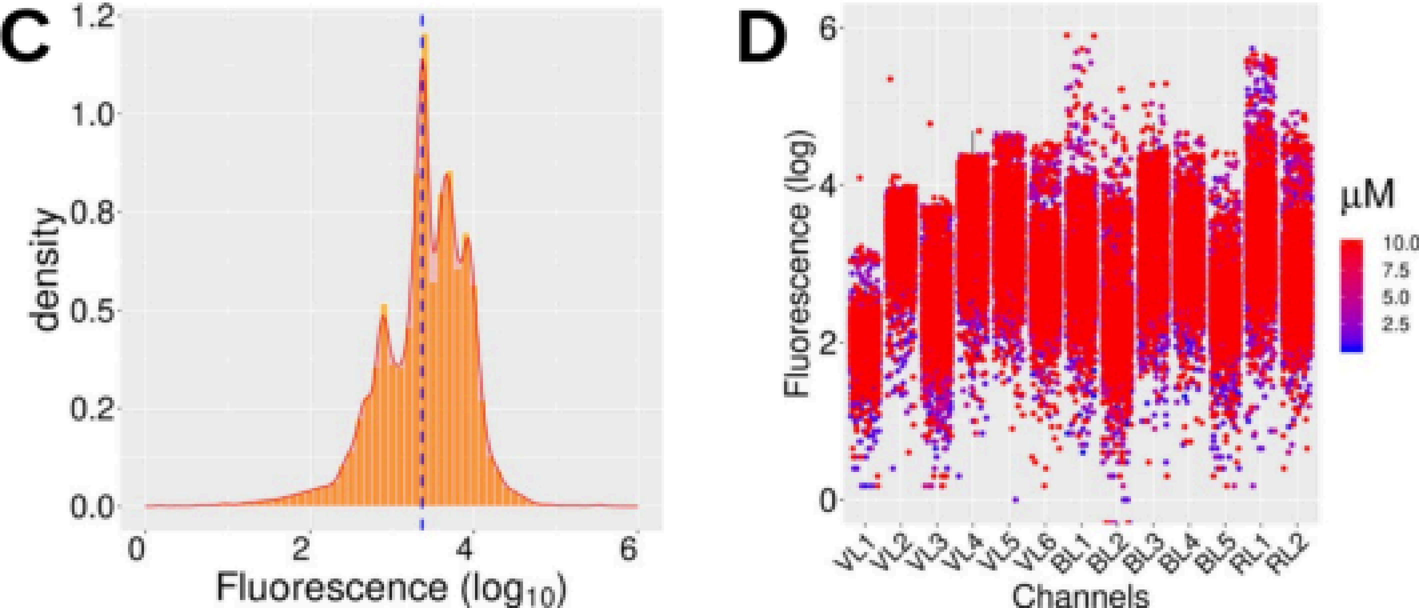

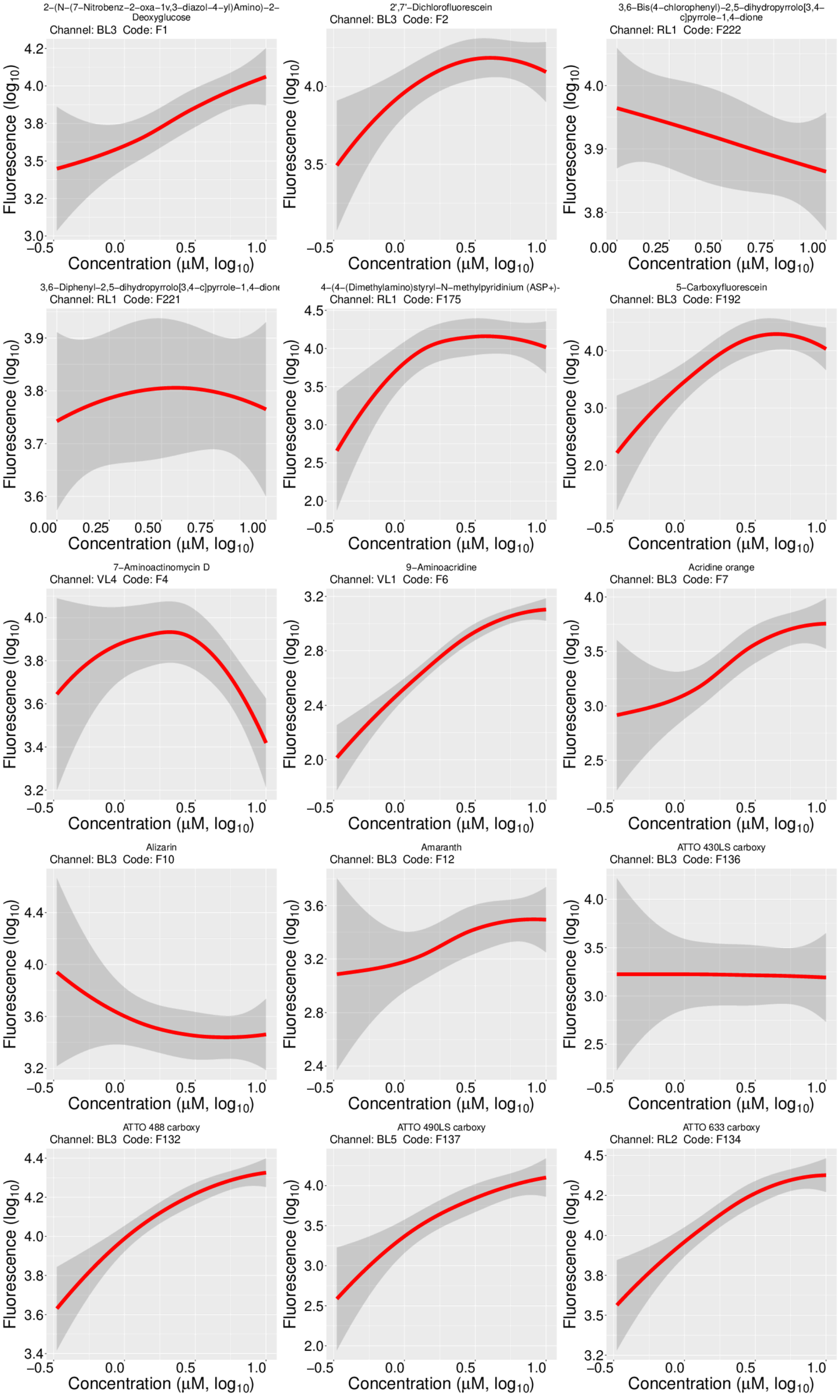
2−(N−(7−Nitrobenz−2−oxa−1v,3−diazol−4−yl)Amino)−2−Deoxyglucose
Channel: BL3 Code: F1
2',7'−Dichlorofluorescein
Channel: BL3 Code: F2
3,6−Bis(4−chlorophenyl)−2,5−dihydropyrrolo[3,4−c]pyrrole−1,4−dione
Channel: RL1 Code: F222
3,6−Diphenyl−2,5−dihydropyrrolo[3,4−c]pyrrole−1,4−dione
Channel: RL1 Code: F221
4−(4−(Dimethylamino)styryl−N−methylpyridinium (ASP+)−
Channel: RL1 Code: F175
5−Carboxyfluorescein
Channel: BL3 Code: F192
7−Aminoactinomycin D
Channel: VL4 Code: F4
9−Aminoacridine
Channel: VL1 Code: F6
Acridine orange
Channel: BL3 Code: F7
Alizarin
Channel: BL3 Code: F10
Amaranth
Channel: BL3 Code: F12
ATTO 430LS carboxy
Channel: BL3 Code: F136
ATTO 488 carboxy
Channel: BL3 Code: F132
ATTO 490LS carboxy
Channel: BL5 Code: F137
ATTO 633 carboxy
Channel: RL2 Code: F134
Fluorescence ($\log_{10}$)
Concentration (μM, $\log_{10}$)

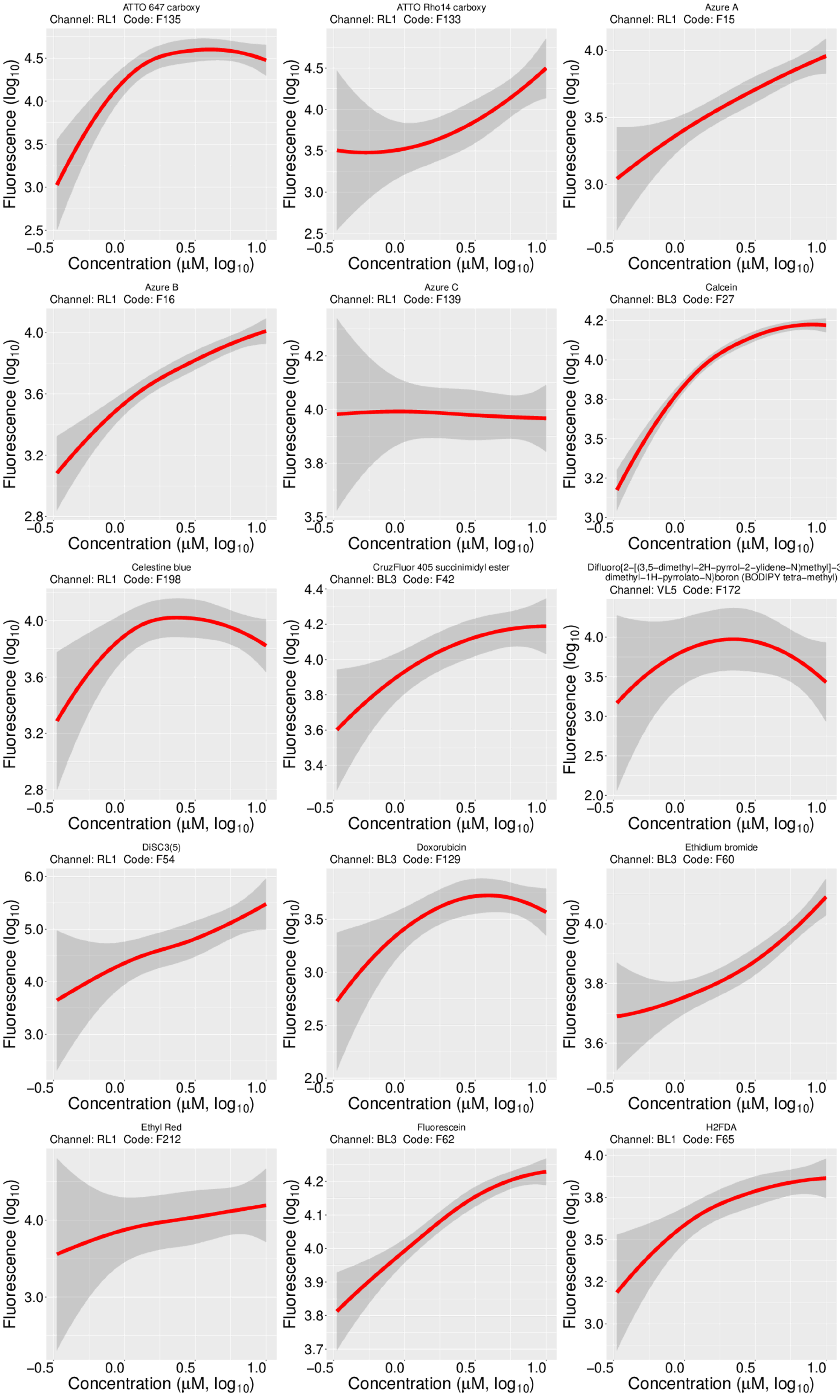
ATTO 647 carboxy
Channel: RL1 Code: F135
Fluorescence (log10)
Concentration (µM, log10)
ATTO Rho14 carboxy
Channel: RL1 Code: F133
Fluorescence (log10)
Concentration (µM, log10)
Azure A
Channel: RL1 Code: F15
Fluorescence (log10)
Concentration (µM, log10)
Azure B
Channel: RL1 Code: F16
Fluorescence (log10)
Concentration (µM, log10)
Azure C
Channel: RL1 Code: F139
Fluorescence (log10)
Concentration (µM, log10)
Calcein
Channel: BL3 Code: F27
Fluorescence (log10)
Concentration (µM, log10)
Celestine blue
Channel: RL1 Code: F198
Fluorescence (log10)
Concentration (µM, log10)
CruzFluor 405 succinimidyl ester
Channel: BL3 Code: F42
Fluorescence (log10)
Concentration (µM, log10)
Difluoro{2–[(3,5–dimethyl–2H–pyrrol–2–ylidene–N)methyl]–3
dimethyl–1H–pyrrolato–N}boron (BODIPY tetra–methyl)
Channel: VL5 Code: F172
Fluorescence (log10)
Concentration (µM, log10)
DiSC3(5)
Channel: RL1 Code: F54
Fluorescence (log10)
Concentration (µM, log10)
Doxorubicin
Channel: BL3 Code: F129
Fluorescence (log10)
Concentration (µM, log10)
Ethidium bromide
Channel: BL3 Code: F60
Fluorescence (log10)
Concentration (µM, log10)
Ethyl Red
Channel: RL1 Code: F212
Fluorescence (log10)
Concentration (µM, log10)
Fluorescein
Channel: BL3 Code: F62
Fluorescence (log10)
Concentration (µM, log10)
H2FDA
Channel: BL1 Code: F65
Fluorescence (log10)
Concentration (µM, log10)

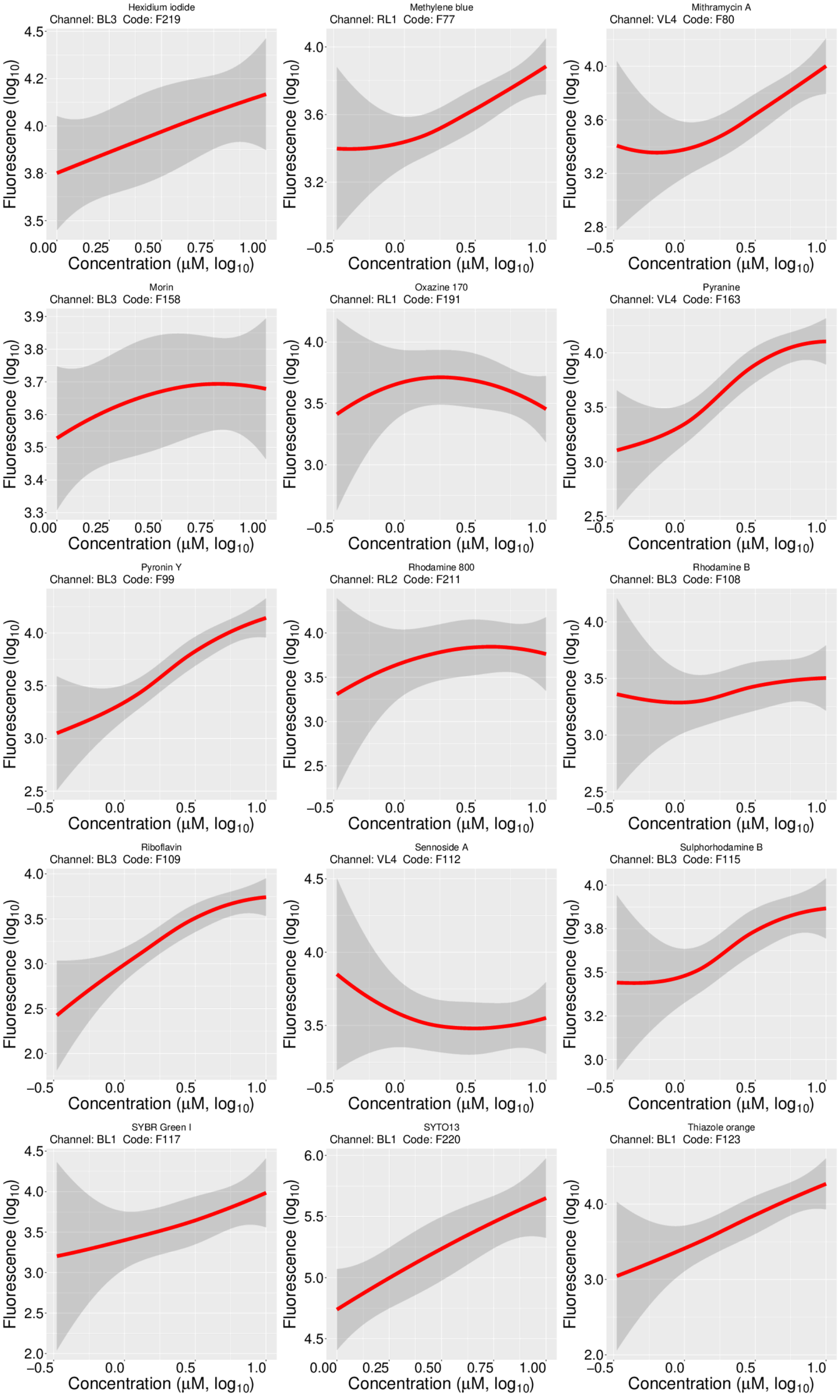
Hexidium iodide
Channel: BL3 Code: F219
Fluorescence (log10)
3.5 3.8 4.0 4.2 4.5
0.00 0.25 0.50 0.75 1.00
Concentration (µM, log10)
Methylene blue
Channel: RL1 Code: F77
Fluorescence (log10)
3.2 3.6 4.0
−0.5 0.0 0.5 1.0
Concentration (µM, log10)
Mithramycin A
Channel: VL4 Code: F80
Fluorescence (log10)
2.8 3.2 3.6 4.0
−0.5 0.0 0.5 1.0
Concentration (µM, log10)
Morin
Channel: BL3 Code: F158
Fluorescence (log10)
3.3 3.4 3.5 3.6 3.7 3.8 3.9
0.00 0.25 0.50 0.75 1.00
Concentration (µM, log10)
Oxazine 170
Channel: RL1 Code: F191
Fluorescence (log10)
3.0 3.5 4.0
−0.5 0.0 0.5 1.0
Concentration (µM, log10)
Pyranine
Channel: VL4 Code: F163
Fluorescence (log10)
2.5 3.0 3.5 4.0
−0.5 0.0 0.5 1.0
Concentration (µM, log10)
Pyronin Y
Channel: BL3 Code: F99
Fluorescence (log10)
2.5 3.0 3.5 4.0
−0.5 0.0 0.5 1.0
Concentration (µM, log10)
Rhodamine 800
Channel: RL2 Code: F211
Fluorescence (log10)
2.5 3.0 3.5 4.0
−0.5 0.0 0.5 1.0
Concentration (µM, log10)
Rhodamine B
Channel: BL3 Code: F108
Fluorescence (log10)
2.5 3.0 3.5 4.0
−0.5 0.0 0.5 1.0
Concentration (µM, log10)
Riboflavin
Channel: BL3 Code: F109
Fluorescence (log10)
2.0 2.5 3.0 3.5 4.0
−0.5 0.0 0.5 1.0
Concentration (µM, log10)
Sennoside A
Channel: VL4 Code: F112
Fluorescence (log10)
3.5 4.0 4.5
−0.5 0.0 0.5 1.0
Concentration (µM, log10)
Sulphorhodamine B
Channel: BL3 Code: F115
Fluorescence (log10)
3.0 3.2 3.5 3.8 4.0
−0.5 0.0 0.5 1.0
Concentration (µM, log10)
SYBR Green I
Channel: BL1 Code: F117
Fluorescence (log10)
2.0 2.5 3.0 3.5 4.0 4.5
−0.5 0.0 0.5 1.0
Concentration (µM, log10)
SYTO13
Channel: BL1 Code: F220
Fluorescence (log10)
4.5 5.0 5.5 6.0
0.00 0.25 0.50 0.75 1.00
Concentration (µM, log10)
Thiazole orange
Channel: BL1 Code: F123
Fluorescence (log10)
2.0 3.0 4.0
−0.5 0.0 0.5 1.0
Concentration (µM, log10)

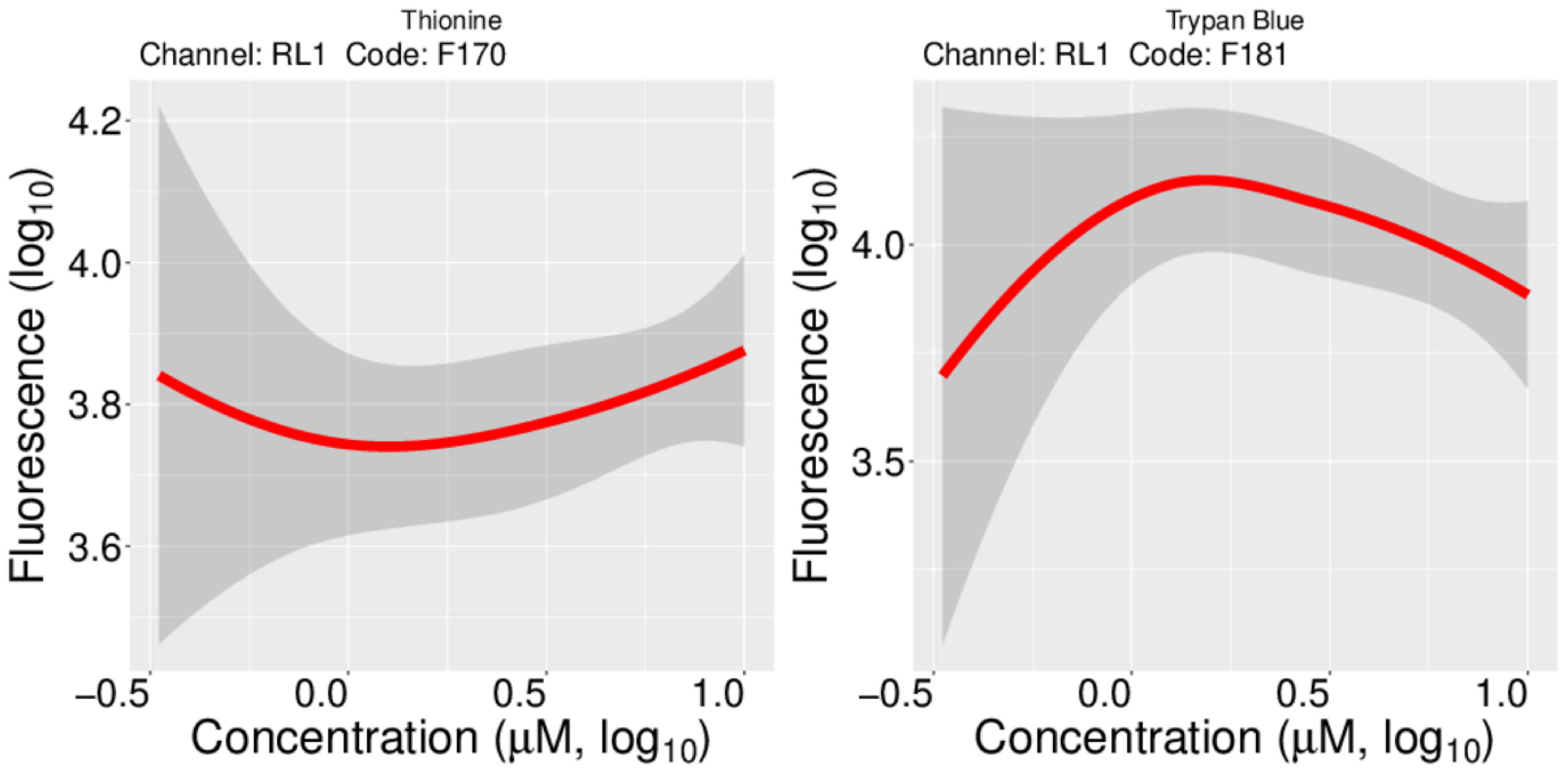

Supplementary Figure 3

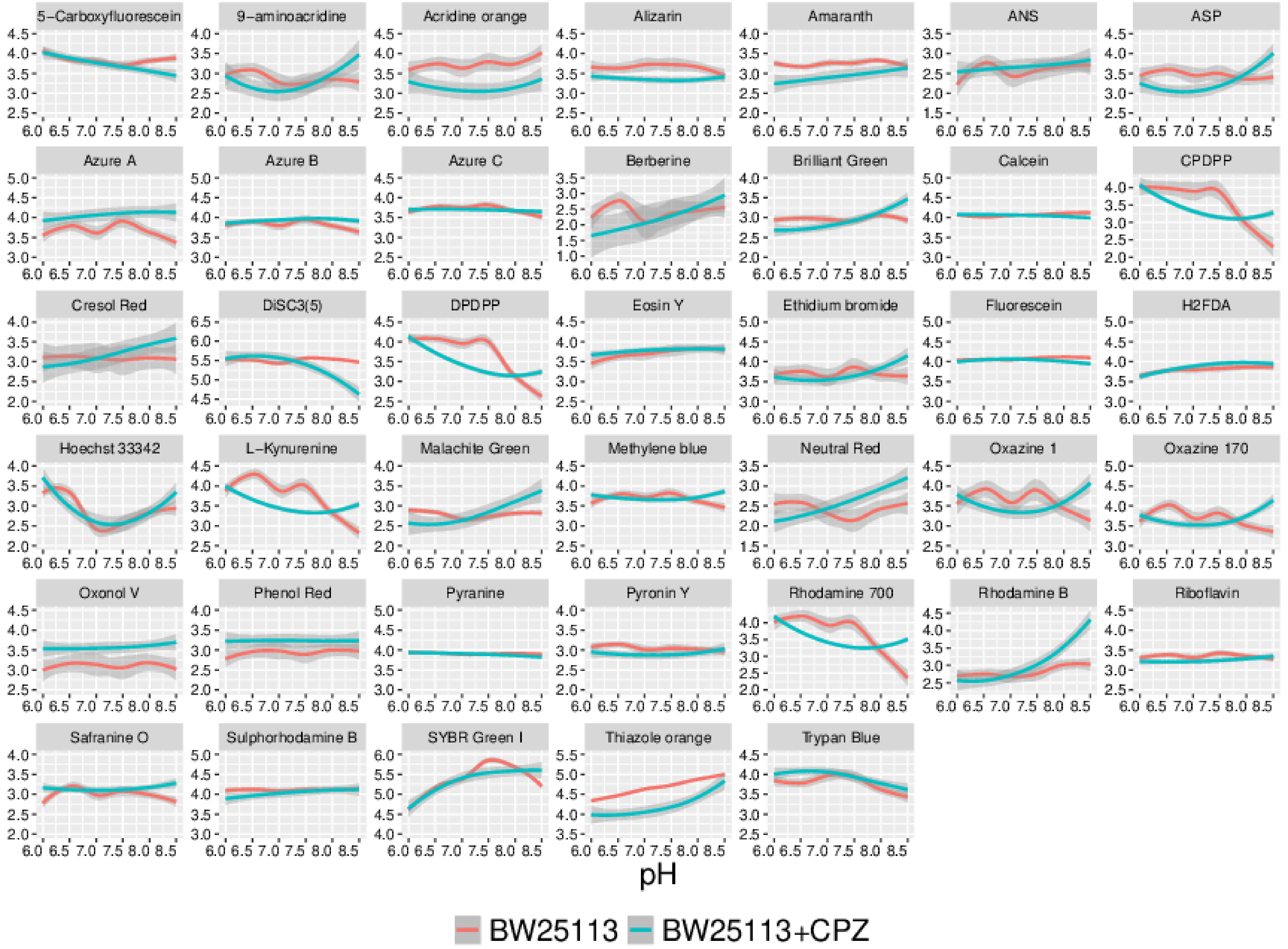
5−Carboxyfluorescein
9−aminoacridine
Acridine orange
Alizarin
Amaranth
ANS
ASP
Azure A
Azure B
Azure C
Berberine
Brilliant Green
Calcein
CPDPP
Cresol Red
DiSC3(5)
DPDPP
Eosin Y
Ethidium bromide
Fluorescein
H2FDA
Hoechst 33342
L−Kynurenine
Malachite Green
Methylene blue
Neutral Red
Oxazine 1
Oxazine 170
Oxonol V
Phenol Red
Pyranine
Pyronin Y
Rhodamine 700
Rhodamine B
Riboflavin
Safranine O
Sulphorhodamine B
SYBR Green I
Thiazole orange
Trypan Blue
pH
BW25113
BW25113+CPZ

Supplementary Figure 5

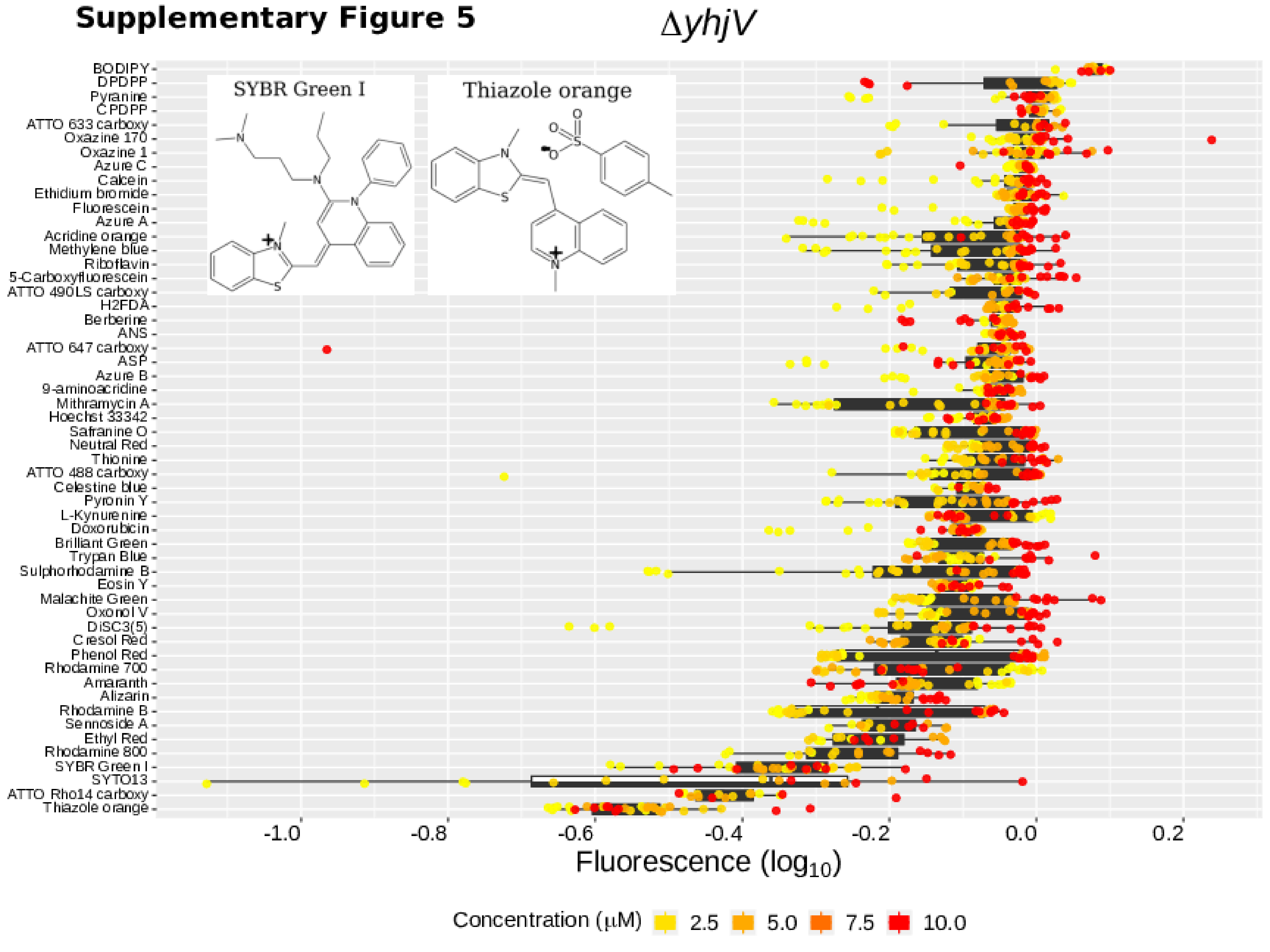

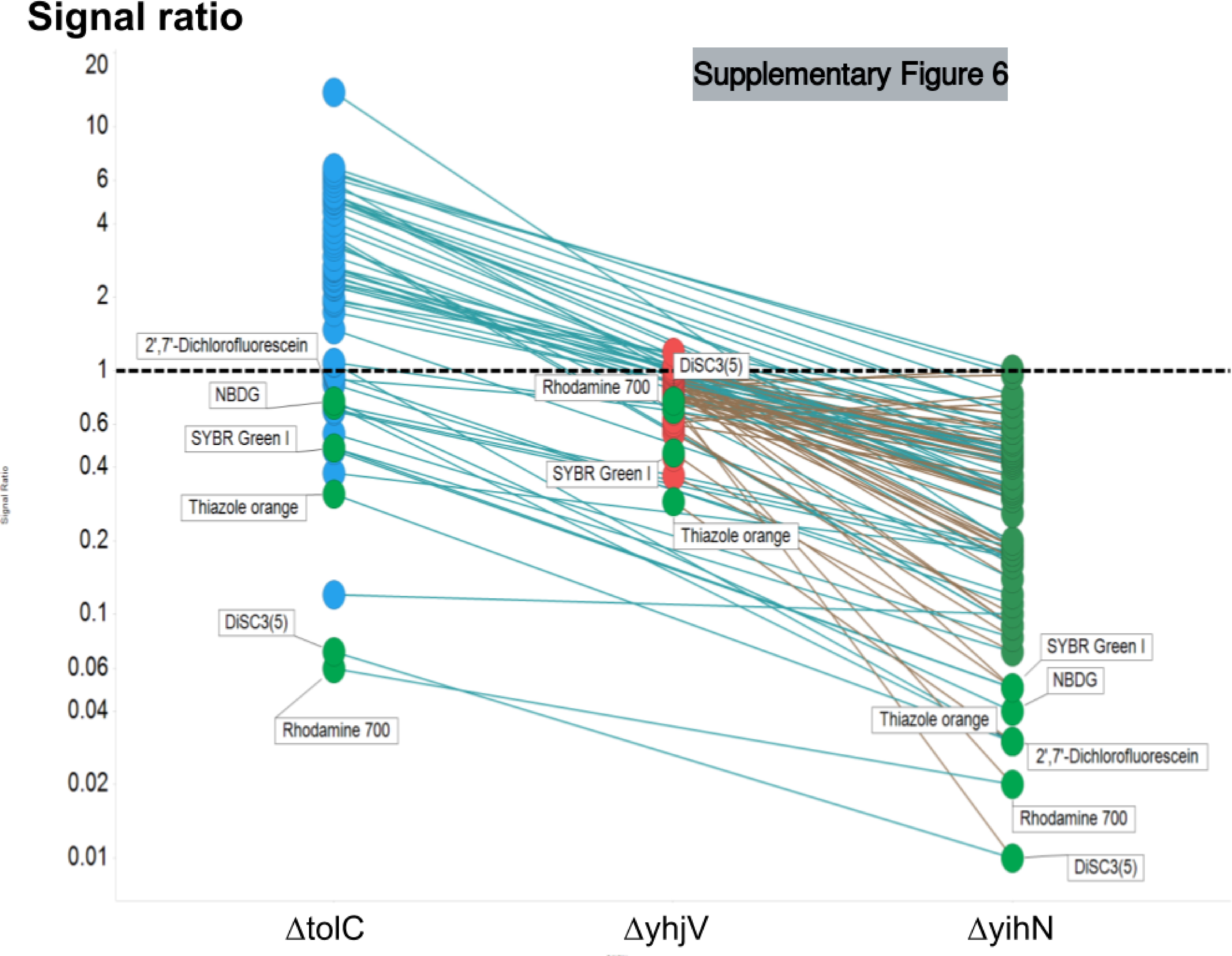
Signal ratio
Supplementary Figure 6
20
10
6
4
2
1
0.6
0.4
0.2
0.1
0.06
0.04
0.02
0.01
2',7'-Dichlorofluorescein
NBDG
SYBR Green I
Thiazole orange
DiSC3(5)
Rhodamine 700
DiSC3(5)
Rhodamine 700
SYBR Green I
Thiazole orange
SYBR Green I
NBDG
Thiazole orange
2',7'-Dichlorofluorescein
Rhodamine 700
DiSC3(5)
ΔtolC
ΔyhjV
ΔyihN
